# Multi-omics and pathway analyses of genome-wide associations implicate regulation and immunity in verbal declarative memory performance

**DOI:** 10.1186/s13195-023-01376-6

**Published:** 2024-01-20

**Authors:** Hao Mei, Jeannette Simino, Lianna Li, Fan Jiang, Joshua C. Bis, Gail Davies, W David Hill, Charley Xia, Vilmundur Gudnason, Qiong Yang, Jari Lahti, Jennifer A. Smith, Mirna Kirin, Philip De Jager, Nicola J. Armstrong, Mohsen Ghanbari, Ivana Kolcic, Christopher Moran, Alexander Teumer, Murali Sargurupremraj, Shamsed Mahmud, Myriam Fornage, Wei Zhao, Claudia L. Satizabal, Ozren Polasek, Katri Räikkönen, David C. Liewald, Georg Homuth, Michele Callisaya, Karen A. Mather, B. Gwen Windham, Tatijana Zemunik, Aarno Palotie, Alison Pattie, Sandra van der Auwera, Anbupalam Thalamuthu, David S. Knopman, Igor Rudan, John M. Starr, Katharina Wittfeld, Nicole A. Kochan, Michael E. Griswold, Veronique Vitart, Henry Brodaty, Rebecca Gottesman, Simon R. Cox, Bruce M. Psaty, Eric Boerwinkle, Daniel I. Chasman, Francine Grodstein, Perminder S. Sachdev, Velandai Srikanth, Caroline Hayward, James F. Wilson, Johan G. Eriksson, Sharon L. R. Kardia, Hans J. Grabe, David A. Bennett, M. Arfan Ikram, Ian J. Deary, Cornelia M. van Duijn, Lenore Launer, Annette L. Fitzpatrick, Sudha Seshadri, Jan Bressler, Stephanie Debette, Thomas H. Mosley

**Affiliations:** 1 Department of Data Science, John D. Bower School of Population Health, University of Mississippi Medical Center, Jackson, MS USA; 2 Gertrude C. Ford Memory Impairment and Neurodegenerative Dementia (MIND) Center, University of Mississippi Medical Center, Jackson, MS USA; 3 Department of Biology, Tougaloo College, Jackson, MS USA; 4 Shanghai Children’s Medical Center, Shanghai Jiao Tong University School of Medicine, Shanghai, China; 5 Department of Medicine, Cardiovascular Health Research Unit, University of Washington, Seattle, WA USA; 6 Department of Psychology, Lothian Birth Cohorts Group, University of Edinburgh, 7 George Square, Edinburgh, EH8 9JZ UK; 7 Icelandic Heart Association, Kopavogur, Iceland; 8 Faculty of Medicine, University of Iceland, Reykjavik, Iceland; 9 Department of Biostatistics, Boston University School of Public Health, Boston, MA USA; 10 The National Heart Lung and Blood Institute’s Framingham Heart Study, Framingham, MA USA; 11 Turku Institute for Advanced Research, University of Turku, Turku, Finland; 12 Department of Psychology and Logopedics, Faculty of Medicine, University of Helsinki, Helsinki, Finland; 13 Department of Epidemiology, School of Public Health, University of Michigan, Ann Arbor, MI USA; 14 Work completed while at The University of Edinburgh, Edinburgh, UK; 15 Taub Institute for Research On Alzheimer’s Disease and the Aging Brain, Columbia Irving University Medical Center, New York, NY USA; 16 Center for Translational and Computational Neuro-Immunology, Columbia University Medical Center, New York, NY USA; 17 Department of Neurology, Columbia University Medical Center, New York, NY USA; 18 Mathematics and Statistics, Curtin University, Perth, Australia; 19 Department of Epidemiology, Erasmus Medical Center University Medical Center, Rotterdam, The Netherlands; 20 School of Medicine, University of Split, Split, Croatia; 21 Department of Geriatric Medicine, Frankston Hospital, Peninsula Health, Melbourne, Australia; 22 Peninsula Clinical School, Central Clinical School, Monash University, Melbourne, Australia; 23 Institute for Community Medicine, University Medicine Greifswald, Greifswald, Germany; 24 Inserm, Bordeaux Population Health Research Center, Team VINTAGE, UMR 1219, University of Bordeaux, Bordeaux, France; 25 The Brown Foundation Institute of Molecular Medicine for the Prevention of Human Diseases, McGovern Medical School, University of Texas Health Science Center at Houston, Houston, TX USA; 26 Human Genetics Center, School of Public Health, University of Texas Health Science Center at Houston, Houston, TX USA; 27 The University of Texas Health Science Center at San Antonio, San Antonio, TX USA; 28 Algebra University College, Ilica 242, Zagreb, Croatia; 29 Interfaculty Institute for Genetics and Functional Genomics, University Medicine Greifswald, Greifswald, Germany; 30 Menzies Institute for Medical Research, University of Tasmania, Hobart, Australia; 31 Centre for Healthy Brain Ageing, School of Psychiatry, University of New South Wales, Sydney, Australia; 32 Neuroscience Research Australia, Sydney, Australia; 33 Department of Medicine, Division of Geriatrics, School of Medicine, University of Mississippi Medical Center, Jackson, MS USA; 34 Department of Medicine, Department of Neurology and Department of Psychiatry, Analytic and Translational Genetics Unit, Massachusetts General Hospital, Boston, MA USA; 35 The Stanley Center for Psychiatric Research and Program in Medical and Population Genetics, The Broad Institute of MIT and Harvard, Cambridge, MA USA; 36 Department of Psychiatry and Psychotherapy, University Medicine Greifswald, Greifswald, Germany; 37 Department of Neurology, Mayo Clinic, Rochester, MN USA; 38 Centre for Global Health Research, Usher Institute, University of Edinburgh, Edinburgh, UK; 39 Alzheimer Scotland Dementia Research Centre, University of Edinburgh, Edinburgh, EH8 9JZ UK; 40 German Center for Neurodegenerative Diseases (DZNE), Site Rostock/ Greifswald, Rostock, Germany; 41 Department of Medicine, School of Medicine, University of Mississippi Medical Center, Jackson, MS USA; 42 Medical Research Council Human Genetics Unit, Institute of Genetics and Cancer, University of Edinburgh, Edinburgh, UK; 43 Dementia Centre for Research Collaboration, University of New South Wales, Sydney, NSW Australia; 44 Stroke, Cognition, and Neuroepidemiology (SCAN) Section, National Institute of Neurological Disorders and Stroke, National Institutes of Health, Bethesda, MD USA; 45 Department of Epidemiology, University of Washington, Seattle, WA USA; 46 Department of Health Services, University of Washington, Seattle, WA USA; 47 Kaiser Permanente Washington Health Research Institute, Seattle, WA USA; 48 Human Genome Sequencing Center, Baylor College of Medicine, Houston, TX USA; 49 Harvard Medical School, Boston, MA USA; 50 Division of Preventive Medicine, Brigham and Women’s Hospital, Boston, MA USA; 51 Department of Medicine, Channing Division of Network Medicine, Brigham and Women’s Hospital, Harvard Medical School, Boston, MA USA; 52 Department of Epidemiology, Harvard T.H Chan School of Public Health, Harvard University, Boston, MA USA; 53 Neuropsychiatric Institute, Prince of Wales Hospital, Sydney, Australia; 54 Department of General Practice and Primary Health Care, University of Helsinki and Helsinki University Hospital, Helsinki, Finland; 55 Department of Public Health Solutions, Chronic Disease Prevention Unit, National Institute for Health and Welfare, Helsinki, Finland; 56 Folkhälsan Research Centre, Helsinki, Finland; 57 Rush Alzheimer’s Disease Center, Rush University Medical Center, Chicago, IL USA; 58 Department of Neurological Sciences, Rush University Medical Center, Chicago, IL USA; 59 Nuffield Department of Population Health, Medical Sciences Division, University of Oxford, Oxford, UK; 60 Laboratory of Epidemiology and Population Sciences, National Institute On Aging, Bethesda, MD USA; 61 Department of Family Medicine, University of Washington, Seattle, WA USA; 62 Glenn Biggs Institute for Alzheimer’s and Neurodegenerative Diseases, San Antonio, TX USA; 63 Department of Neurology, Boston University School of Medicine, Boston, MA USA; 64 Department of Neurology, CHU de Bordeaux, Bordeaux, France; 65 Department of Neurology, University of Mississippi Medical Center, Jackson, MS USA

**Keywords:** Genome-wide association study, Memory, Expression, Immunity, Multi-omics, Delayed recall

## Abstract

**Background:**

Uncovering the functional relevance underlying verbal declarative memory (VDM) genome-wide association study (GWAS) results may facilitate the development of interventions to reduce age-related memory decline and dementia.

**Methods:**

We performed multi-omics and pathway enrichment analyses of paragraph (PAR-dr) and word list (WL-dr) delayed recall GWAS from 29,076 older non-demented individuals of European descent. We assessed the relationship between single-variant associations and expression quantitative trait loci (eQTLs) in 44 tissues and methylation quantitative trait loci (meQTLs) in the hippocampus. We determined the relationship between gene associations and transcript levels in 53 tissues, annotation as immune genes, and regulation by transcription factors (TFs) and microRNAs. To identify significant pathways, gene set enrichment was tested in each cohort and meta-analyzed across cohorts. Analyses of differential expression in brain tissues were conducted for pathway component genes.

**Results:**

The single-variant associations of VDM showed significant linkage disequilibrium (LD) with eQTLs across all tissues and meQTLs within the hippocampus. Stronger WL-dr gene associations correlated with reduced expression in four brain tissues, including the hippocampus. More robust PAR-dr and/or WL-dr gene associations were intricately linked with immunity and were influenced by 31 TFs and 2 microRNAs. Six pathways, including type I diabetes, exhibited significant associations with both PAR-dr and WL-dr. These pathways included fifteen MHC genes intricately linked to VDM performance, showing diverse expression patterns based on cognitive status in brain tissues.

**Conclusions:**

VDM genetic associations influence expression regulation via eQTLs and meQTLs. The involvement of TFs, microRNAs, MHC genes, and immune-related pathways contributes to VDM performance in older individuals.

**Supplementary Information:**

The online version contains supplementary material available at 10.1186/s13195-023-01376-6.

## Background

Delayed verbal declarative memory (VDM) performance, commonly measured by paragraph and word list delayed recall tests, is an important predictor of Alzheimer’s disease (AD) [1]. Genome-wide association studies (GWAS) have leveraged VDM performance (heritability≈30–52% [2, 3]) to identify variants influencing brain aging and AD susceptibility. The largest such GWAS, led by the Cohorts for Heart and Aging Research in Genomic Epidemiology (CHARGE) Cognitive Working Group, identified three significant chromosomal regions (near *APOE*,* HS3ST4*, and *SPOCK3*) in a sample of 29,076 older non-demented participants of European descent [2]. A genetic risk score combining fifty-eight independent suggestive variants was associated with AD pathology (neurofibrillary tangle density and amyloid plaque burden) in autopsy samples [2], demonstrating that genetic studies of VDM can provide insight into the molecular contributors to AD pathobiology.

GWAS often implicate non-coding regions suspected to influence regulation [4], lack power to detect the small effect sizes bestowed by most genetic variants [5], are encumbered by the heterogeneity of genetic effects across studies [6], and have severe multiple testing corrections [5, 7, 8]. The integration of additional biological resources and aggregation of effects across genes and pathways can address these limitations and facilitate the interpretation of GWAS results [9] to understand biological functions [4]. Both multi-omics and pathway analyses can integrate GWAS findings with functional information from publicly available databases to gain insight into complex trait pathobiology [9] and provide context to interpret genotype–phenotype relationships [4].

Debette et al. identified a VDM-associated genetic variant in proximity to genes linked to immune responses [2]. Additionally, they found that variants associated with suggestive memory risks correlate with gene expressions in human hippocampus samples. Building upon these findings, our study endeavors to expand beyond the limitations of prior research by delving into the potential functions associated with VDM-related genetic variants. To achieve this, we employed multi-omics analyses to explore the intricate relationship between VDM-associated genetic variants and expression quantitative trait loci (eQTLs), methylation quantitative trait loci (meQTLs), and gene expressions across diverse tissue types. Our investigation also meticulously examined how the associations of genetic variants with VDM are intertwined with the regulatory activities of transcription factors (TFs) and microRNAs, along with immune gene functions. Additionally, we undertook the task of evaluating the genetic pathways that underlie the associations related to paragraph delayed recall (PAR-dr) and word list delayed recall (WL-dr)[4], along with exploring links between pathway gene expressions and cognitive status in brain tissues.

## Methods

### Participating cohorts and phenotypes

This study utilized data from twenty-seven cohorts comprising individuals of Caucasian descent, divided into 19 for the initial discovery phase and 8 for replication. The dataset included HapMap-imputed genome-wide single-nucleotide polymorphism (SNP) data, and at least one test of PAR-dr or WL-dr. Consequently, we conducted the analyses in this study using summarized data from a prior GWAS meta-analysis that specifically focused on PAR-dr and WL-dr within these cohorts [2]. Detailed information about these cohorts can be found in the Supplemental Text and Tables S1 and S2.

Participants provided written informed consent and all studies were approved by their respective institutional review boards. The nineteen discovery cohorts (8 for PAR-dr and 15 for WL-dr) collectively represented 29,076 (N_PAR- dr_ = 6674; N_WL-dr_ = 24,604) dementia- and stroke-free Caucasian participants aged 45 years or older (Figure S1). The eight replication cohorts represented approximately 8000 (N_PAR- dr_ = 8009; N_WL-dr_ = 7518) stroke-free Caucasian participants aged 65 years or older; dementia assessment was not universally available in the replication cohorts and seven of the eight replication cohorts were restricted to women with some college education.

For the PAR-dr tests, participants were verbally presented one or two stories and asked to recall as many paragraph elements as possible after a 20- or 30-min delay and an interceding immediate recall task. For the WL-dr tests, participants were verbally or visually presented a list of semantically related or unrelated words (10–16 words over 1–5 exposure trials) and asked to recall as many words as possible after a 3- to 30-min delay and an interceding immediate recall task. The outcomes were the total number of items recalled during the delayed recall tasks.

### Cohort-specific genetic associations

#### Single-variant associations

Separate GWAS analyses were performed for PAR-dr and WL-dr within each cohort; the cohort-specific summary results for each trait were obtained from the CHARGE consortium. Within each cohort, a linear regression model of the number of story elements or words recalled was fit onto the number of minor alleles at each SNP while adjusting for age and sex, as well as study site, familial structure, and population substructure if necessary [2]. Subsequently, single-variant associations from each participating cohort were gathered for further analysis.

#### Gene associations

We measured gene associations from independent SNPs in each cohort. GWAS SNPs (≈1.5 to 2.4 million per GWAS) were mapped to genes (≈35,000 to 38,000 including non-RNA coding genes) using 2 kb upstream/downstream boundaries of the transcription start/stop sites (Tables S1 and S2), referencing genome Build GRCh37. Within each gene, pairwise SNP correlation coefficients (*r*^2^) were calculated using VCFtools [10] and the European reference data from the 1000 Genomes project. Clumping was conducted to select independent SNPs through an iterative process; at each step, we selected the SNP with the strongest association and removed SNPs correlated *(r*^*2*^ > 0.2*)* to it.

We computed Simes’ combination *p*-value of gene [11] as $M={\text{min}}(k\bullet {p}_{\left(i\right)}/i)$, where *k* was the number of total independent SNPs and *p*_(*i*)_ was the *i*th smallest *p*-value. Gene uniform-score (*U*-score) [12] was applied to measure gene association and it was calculated as $=({\sum }_{j=1}^{L}I\left({M}_{j}<M\right)+0.5{\sum }_{j=1}^{L}I\left({M}_{j}=M\right))/L$, where ${M}_{j}$ was the combination *p*-value of the *j*th gene and *L* is the total number of genes. Gene *U*-score ranges from zero to one, and it estimates the proportion of genes with a stronger association than the tested gene. Genes with *U*-scores ≤ 0.05 were selected as phenotype-associated genes.

### Meta-analysis of genetic associations

#### Single-variant associations

We employed METAL [13] to conduct a sample-size weighted meta-analysis for each phenotype (PAR-dr and WL-dr) and genetic variant across the discovery cohorts alone and the discovery and replication cohorts together.

#### Gene associations

For each gene, we counted the number of cohorts with *U*-scores less than or equal to 0.05. Meta-analysis *p*-value of each gene (Gene_p) was computed from binomial distribution and Bonferroni-corrected significance threshold was set as 1E − 06 (0.05/50,000 to adjust for 50,000 genes tested).

### Multi-omics function analyses

**Fig. 1:**
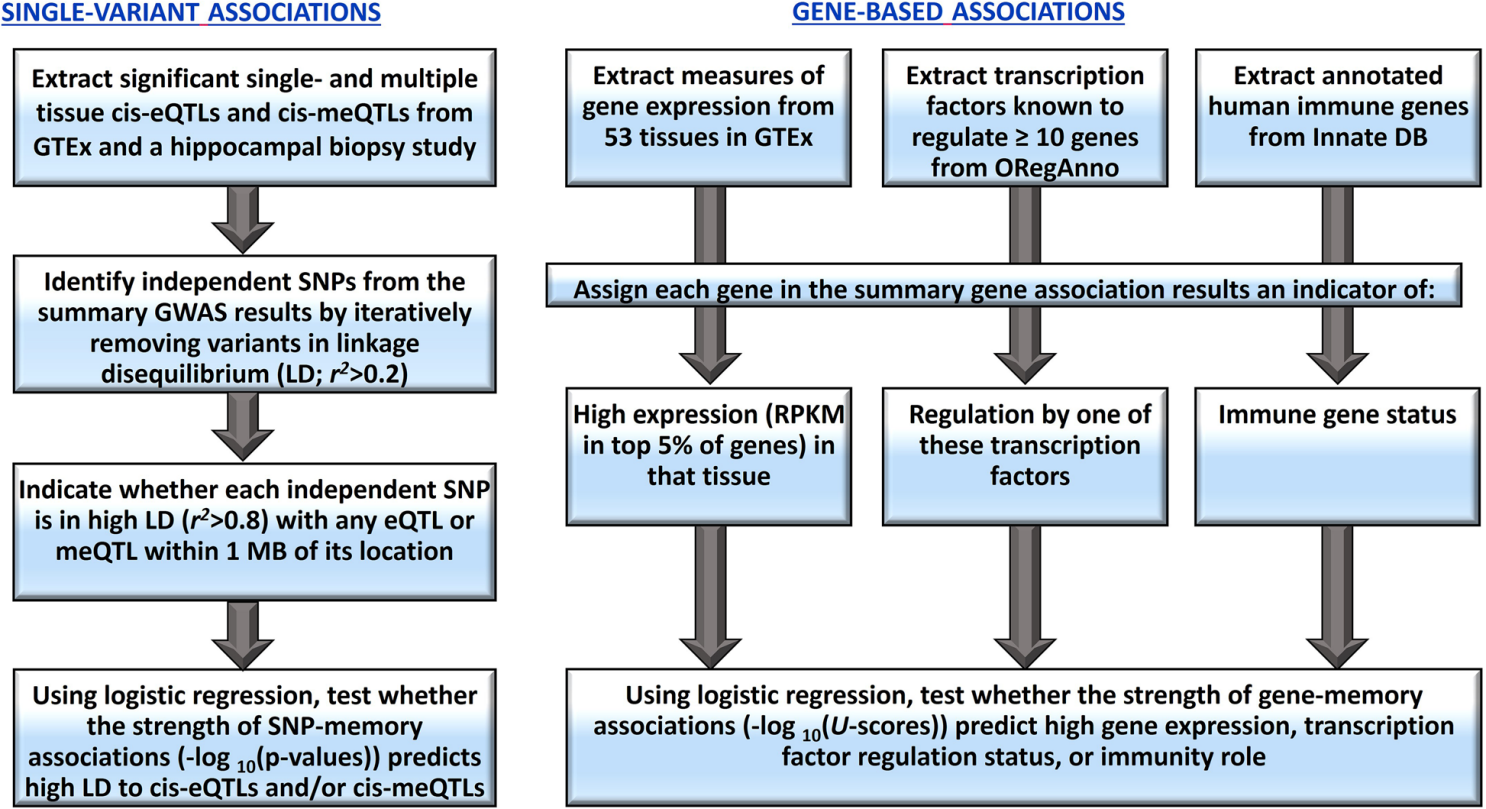
Design of the multi-omics analyses

The overall design of the multi-omics function analyses for single-variant and gene associations is depicted in Fig. 1.

#### Functions of single-variant associations

We employed logistic regression to evaluate the relationship between VDM-associated genetic variants and eQTLs and meQTLs across different tissues. We extracted significant cis-eQTLs within ± 1 MB of transcription start sites from 44 different tissues of the GTEx Project [14]. We similarly extracted significant eQTLs and meQTLs from a genome-wide study of 110 human hippocampal biopsies [15]. We identified independent SNPs from meta-analysis of discovery cohorts and examined their LD status with eQTLs (and meQTLs for the hippocampal biopsy data) from each tissue alone and all tissues combined. The LD status indicated whether the SNP was in high LD (*r*^*2*^ ≥ 0.8) with any eQTL or meQTL within 1 MB. We performed logistic regression of the LD status on the negative log base-10 of the single-variant association *p*-values in each tissue and all tissues combined. We conducted 10,000 permutations to adjust for multiple tests; permutation *p*-values ≤ 0.05 were considered significant.

#### Functions of gene associations

We utilized logistic regression to investigate potential links between VDM gene associations and gene expression, immune function, and transcription factor (TF) and microRNA regulation. We extracted GTEx gene expression, measured as reads per kilobase per million reads (RPKM), from 53 tissues via UCSC genome browser [16]. A gene was highly expressed if its RPKM ranked in the top 5% of all genes for that tissue. We extracted 41 TFs and 52 microRNAs regulating at least ten genes from the Open Regulatory Annotation database (ORegAnno) [17]. TF regulation for a gene was identified if it was regulated by the TF/microRNA. Lastly, the immunity function of a gene was identified if it was annotated as a human immune gene in the InnateDB [18]. We fitted logistic models of status of gene expression, TF regulation, and immune function onto the − log_10_ *U*-scores for the gene association. An adjusted *p*-value ≤ 0.05 was considered significant, based on 1000 permutation tests.

### Pathway enrichment of genetic associations

#### Cohort-specific pathway associations

Gene set enrichment analyses were performed to examine VDM-associated pathways based on cohort-specific GWAS of PAR-dr and WL-dr. We employed the uniform-score gene-set analysis (USGSA) method [12] to test pathways enriched for genes with *U*-scores ≤ 0.05 among 10,295 curated gene sets from the MSigDB knowledge base [19] in every cohort. Pathway enrichment analysis was conducted using the R package of snpGeneSets [20]. For a MSigDB gene set ($\Omega$) and a set of genes ($\Phi$) with *U*-scores ≤ 0.05, the probability that a component gene of $\Omega$(${G}_{i}$) belongs to $\Phi$ is defined as ${p}_{\Omega }={\text{Pr}}\left({G}_{i}\in\Phi |{G}_{i}\in\Omega \right)$ and estimated as $\widehat{{p}_{\Omega }}=\frac{{\sum }_{i}I({G}_{i}\in \Omega \bigcap {G}_{i}\in \Phi )}{{\sum }_{i}I({G}_{i}\in\Omega )}$. In contrast, ${p}_{0}=0.05$ is the null probability of a random gene (${G}_{i}$) belonging to $\Phi$. The pathway enrichment effect, $E=\widehat{{p}_{\Omega }}-{p}_{0}$, shows the increased probability of a pathway component gene (versus a random gene) to have a *U*-score ≤ 0.05, and the standard error (SE) is estimated as $SE=\sqrt{{p}_{0}\cdot (1-{p}_{0})/{\sum }_{i}I({G}_{i}\in\Omega )}$. The pathway exact *p*-value was calculated from the hypergeometric distribution; we adjusted for multiple testing and correlations due to genes belonging to multiple pathways by 10,000 permutations, yielding the adjusted *p*-value (path_p_k_) in the *k*th cohort.

#### Meta-analysis of pathway enrichment over cohorts (Approach 1)

**Fig. 2:**
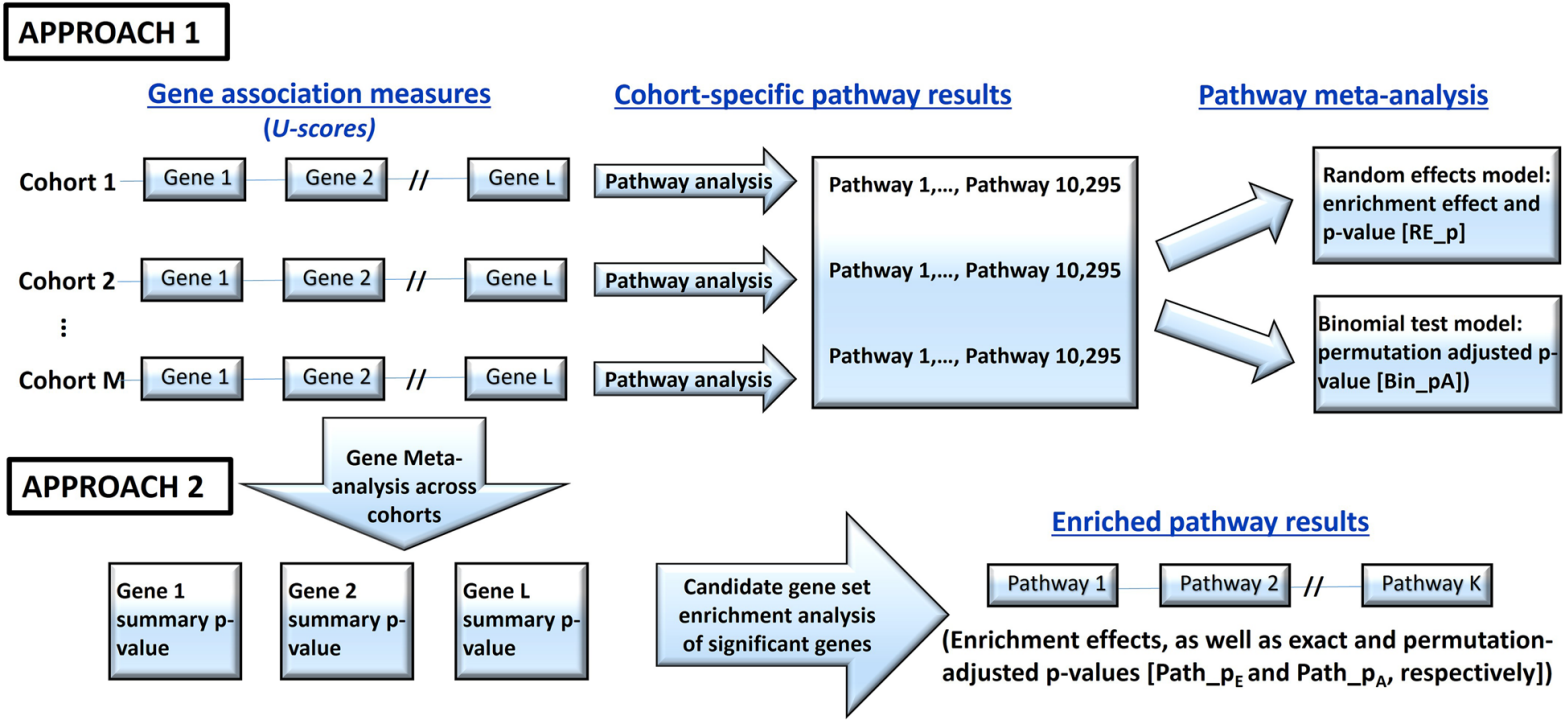
Pictorial representation of the two approaches used to derive the overall pathway results from the cohort-specific genome-wide associations

Two meta-analyses, random-effects (RE) model and the binomial test, were employed to estimate the effects of pathway enrichment across different cohorts and to ascertain whether the occurrence of VDM-associated pathways in the participating cohorts exhibited a non-random pattern (Figs. 2 and S2). Both meta-analyses were performed in the discovery cohorts alone, the replication cohorts alone, and all cohorts combined. The RE meta-analysis, performed using the *R* package metafor [21], incorporated the inverse variance of the effect estimate as a cohort weight. The RE model produced a summary enrichment effect estimate and a *p*-value (RE_p) of tested gene set over cohorts. The significance threshold for RE_p in the meta-analysis of discovery cohorts alone and the discovery and replication cohorts combined was 4.86E − 06 after Bonferroni correction (0.05/10,295). In the replication cohorts, a Bonferroni correction accounted for the number of pathways tested.

The binomial test was applied to count the number of cohorts with significant pathway enrichment and compute the exact *p*-value from binomial distribution (Supplemental Text). For the discovery cohorts alone and the discovery and replication cohorts combined, the binomial test was based on permutation-adjusted pathway *p*-values (path_p_k_) from individual cohorts and *p*-value (Bin_p_A_) ≤ 0.05 was considered significant. For replication cohorts alone, the *p*-value (Bin_p) was based on pathway *p*-value from individual cohorts and Bonferroni adjustment was adopted.

#### Pathway enrichment of significant genes over cohorts (Approach 2)

Significant genes with meta-analysis *p*-values (i.e., Gene_*p* ≤ 1E − 06) were selected and tested for enrichment in a particular MSigDB gene set. The exact pathway *p*-value (Path_p_E_) was calculated from the hypergeometric distribution; pathway *p*-value (Path_p_A_) adjusted for multiple testing was obtained via 10,000 permutations with significance threshold of 0.05.

### Differential expression (DE) analysis of significant pathway component genes

We performed DE analyses using significant component genes (Gene_*p* ≤ 1E-06) from VDM-associated pathways. Three curated human (GDS4135 [22], GDS4231 [23], GDS4358 [24]) and rodent (GDS2082 [25], GDS2639 [26], GDS520 [27]) gene expression studies of cognitive traits were selected from the Gene Expression Omnibus [28]; descriptions of each study are provided in the Supplemental Text. The rodent studies used homologs (identified through the NCBI HomoloGene tool [29]) in hippocampal tissue.

For both human and rodent studies, the gene expression values were normalized by quantile normalization using the R package preprocessCore [30]. We used linear models from the R package limma [31] to analyze the DE of each gene across cognitive statuses; an F statistic and *p*-value were generated after moderating the test standard errors by empirical Bayesian modeling. The gene-set DE test was based on designed contrast tests for comparing expression levels by cognition status and utilized the mean-rank method [32] implemented in limma. *P*-values were obtained through permutation tests, with significance defined as *p*-values ≤ 0.05.

## Results

### Multi-omics function analysis of single-variant associations

**Fig. 3:**
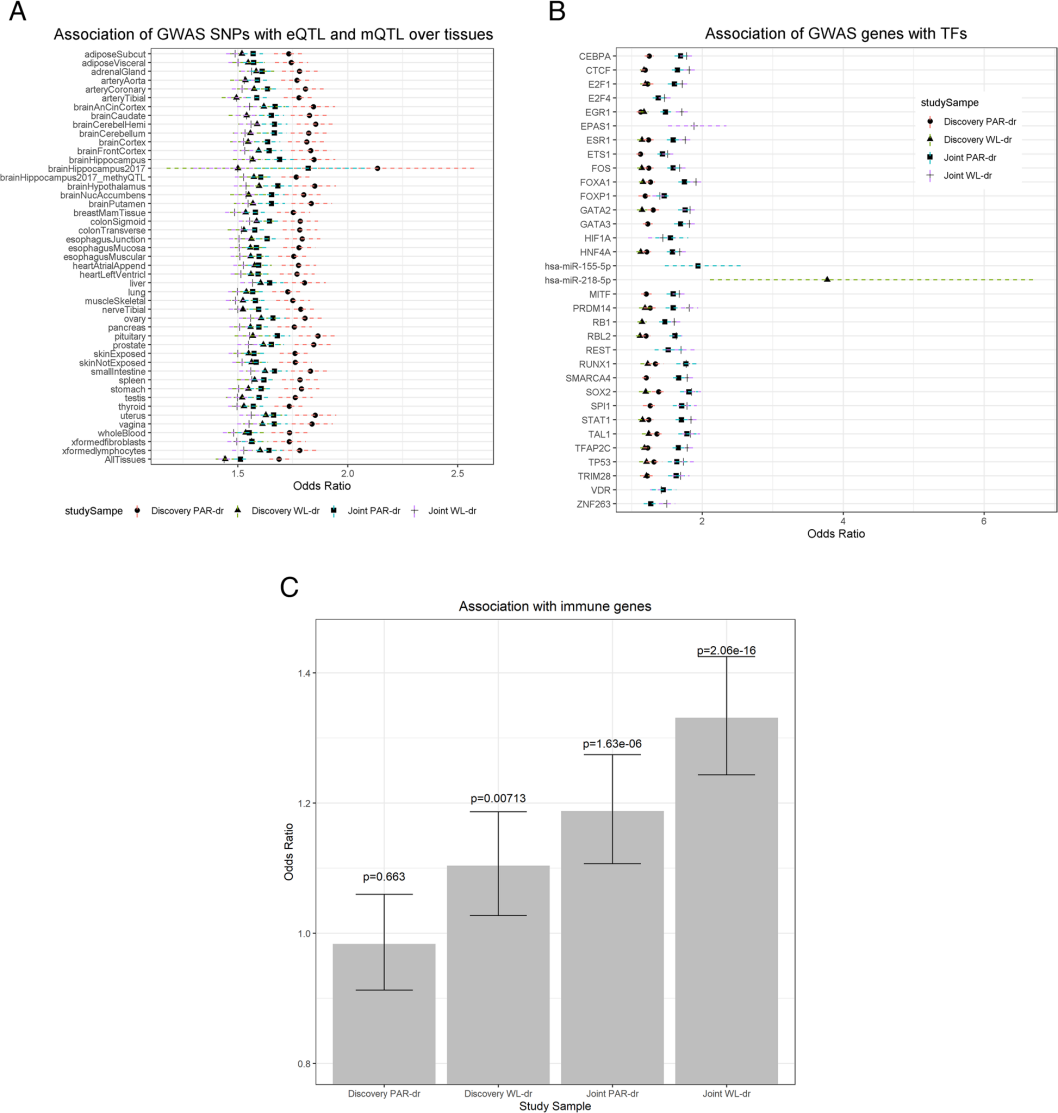
**A** Relationship between the strength ((− log 10 (*p*-values)) of verbal declarative memory single-variant associations and being in high linkage disequilibrium (*r*^2^ > 0.80) with eQTLs and meQTLs across tissues. The shapes with dotted lines represent the odds ratios of being in linkage disequilibrium with an eQTL or meQTL given a one unit increase in SNP-memory association significance (*p*-value decreasing by a power of 10). The length of dotted line denotes the 95% confidence intervals of the odds ratios. **B** Relationship between the strength ((− log 10 (*U*-score)) of verbal declarative memory gene associations and regulation by known transcription factors and microRNAs. The shapes with dotted lines represent the odds ratios of being regulated by a transcription factor or microRNA given a one unit increase in gene association significance (*U*-score decreasing by a power of 10). The length of dotted line denotes the 95% confidence intervals of the odds ratios. **C** Relationship between the strength ((− log 10 (*U*-score)) of verbal declarative memory gene associations and annotation as an immunity gene. The heights of the bars represent the odds ratios of being an annotated immune gene given a one unit increase in gene association significance (*U*-score decreasing by a power of 10). The bars denote the 95% confidence intervals of the odds ratios

Cross-cohort single-variant memory associations were related to markers of regulation (eQTLs and meQTLs) as shown in Fig. 3A and Table S3. Regardless of the tissue tested, variants highly associated with VDM phenotypes had significantly greater odds of being in high LD with eQTLs and meQTLs; the odds ratio (OR) estimates ranged from 1.43 (*β* = 0.36) to 2.14 (*β* = 0.76). Each power of 10 increase in association (e.g. *p*-value decreasing from 1E − 05 to 1E − 06) corresponded to at least a 1.43 increase in the odds of being in high LD with an eQTL or meQTL. The OR of PAR-dr single-variant associations exceeded those of WL-dr. The largest OR (2.14; 95% CI [1.76, 2.60]) corresponded to the effect of PAR-dr single-variant associations on eQTLs from hippocampal biopsies in discovery cohorts, with an OR of 1.82 (95% CI [1.59, 2.09]) in the discovery and replication cohorts combined.

### Multi-omics function analysis of gene associations

VDM gene associations were implicated in gene expression, regulation by TF/microRNA, and immunity function. As shown in Table 1, genes more strongly associated with WL-dr exhibited decreased odds of being highly expressed (RPKM in the top 5%) in four brain tissues, namely the anterior cingulate cortex, caudate, hippocampus, and pituitary gland. For the former three tissues, the negative association is significant in the discovery cohorts. For the pituitary gland, the negative association is significant in the joint discovery and replication cohorts. We failed to detect any significant relationship between PAR-dr gene associations and expression.

**Table 1:** Significant tissue-specific correlation between GWAS associations and gene expression

Tissue	Summary gene result	WL-dr			PAR-dr		
		**OR**	**95% CI**	**Permutation-adjusted *p*-value**	**OR**	**95% CI**	**Permutation adjusted *p*-value**
Brain anterior cingulate cortex	Discovery	0.75	[0.64,0.87]	**0.005**	1.00	[0.88,1.15]	1.000
	Joint	0.86	[0.75,0.99]	0.399	0.96	[0.84,1.10]	1.00
Brain caudate	Discovery	0.78	[0.68,0.91]	**0.026**	1.03	[0.90,1.17]	1.000
	Joint	0.91	[0.79,1.04]	0.897	0.98	[0.86,1.12]	1.000
Brain hippocampus	Discovery	0.80	[0.69,0.92]	**0.046**	0.99	[0.87,1.13]	1.000
	Joint	0.89	[0.77,1.02]	0.725	0.93	[0.81,1.07]	0.997
Pituitary	Discovery	0.90	[0.78,1.03]	0.838	0.93	[0.81,1.07]	0.998
	Joint	0.78	[0.68,0.90]	**0.021**	0.85	[0.74, 0.98]	0.350

Genes more strongly associated with VDM had significantly increased odds of being regulated by thirty-one TFs and two microRNAs (Fig. 3B and Table S4); thirty TFs were implicated for both PAR-dr and WL-dr using all cohorts. Their ORs ranged from 1.12 (95% CI [1.06, 1.18]) for *RBL2* to 3.78 (95% CI [2.10, 6.81]) for hsa-miR-218-5p (95% CI [2.10, 6.81]), both of which were observed in the discovery WL-dr. The ORs were larger analyzing all cohorts than discovery cohorts alone with one exception, WL-dr gene associations and hsa-miR-218-5p. Similarly, genes with stronger VDM associations had greater odds of being immune genes. Both PAR-dr (OR = 1.19, 95% CI [1.11, 1.27]) and WL-dr (OR = 1.33,95% CI [1.24, 1.43]) gene associations were significantly related to immune gene functions when analyzing all cohorts (Fig. 3C).

### Pathway enrichment analysis

#### Meta-analysis of pathway enrichment over cohorts (Approach 1)

Six pathways, namely the set of genes upregulated with PSMD4 and the KEGG pathways of type I diabetes mellitus, graft-versus-host disease, allograft rejection, antigen processing and presentation, and viral myocarditis, were significantly (*p*-values: RE_*p* ≤ 4.86E − 06 or Bin_p_A_ ≤ 0.05) associated with PAR-dr and WL-dr in discovery cohorts (Table 2). The enrichment effect sizes (12 ~ 28%) were similar for PAR-dr and WL-dr in discovery cohorts; forest plots of the enrichment effects for each pathway and trait are displayed in Figure S3.

**Table 2:** Significant pathways identified by Approach 1 (meta-analysis of cohort-specific pathway enrichment effects and tests)

	**Meta discovery**							
	**PAR-dr**				**WL-dr**			
***Gene set (size)***	***Effect***	***SE***	***RE_p***	***Bin_p***_***A***_	***Effect***	***SE***	***RE_p***	***Bin_p***_***A***_
Type 1 diabetes (44)	20.94%	1.22%	3.32E − 66	5.79E − 03	22.67%	1.72%	7.59E − 40	2.32E − 10
*PSMD4* targets (73)	14.33%	1.11%	7.24E − 38	5.79E − 03	15.40%	1.20%	8.44E − 38	1.83E − 07
Graft-versus-host disease (42)	24.26%	1.69%	1.60E − 46	1.54E − 05	25.60%	2.07%	4.17E − 35	2.32E − 10
Allograft rejection (38)	25.57%	1.34%	3.98E − 81	4.01E − 07	27.73%	1.99%	3.28E − 44	7.42E − 09
Antigen processing and presentation (89)	12.53%	0.88%	1.52E − 46	5.79E − 03	13.05%	1.31%	1.70E − 23	3.52E − 06
Viral myocarditis (73)	11.76%	0.94%	6.97E − 36	> 0.05	13.51%	1.04%	1.05E − 38	5.28E − 05
	**Meta replication**							
	**PAR-dr**				**WL-dr**			
***Gene set***	***Effect***	***SE***	***RE_p***	***Bin_p*ǂ**	***Effect***	***SE***	***RE_p***	***Bin_p*ǂ**
Type 1 diabetes	0.63%	1.47%	0.67	1.00	3.44%	1.83%	0.060	0.006
*PSMD4* targets	0.39%	1.01%	0.70	1.00	1.88%	0.94%	0.046	0.34
Graft-versus-host disease	− 0.48%	1.28%	0.71	1.00	2.64%	1.36%	0.053	0.34
Allograft rejection	0.28%	1.34%	0.83	1.00	1.42%	1.76%	0.42	0.34
Antigen processing and presentation	− 1.25%	0.86%	0.15	1.00	2.19%	1.56%	0.16	0.34
Viral myocarditis	0.40%	1.13%	0.72	0.34	1.52%	1.44%	0.29	0.057
	**Meta joint**							
	**PAR-dr**				**WL-dr**			
***Gene set***	***Effect***	***SE***	***RE_p***	***Bin_p***_***A***_	***Effect***	***SE***	***RE_p***	***Bin_p***_***A***_
Type 1 diabetes	10.78%	2.77%	1.01E − 04	0.04	15.98%	2.32%	6.23E − 12	6.11E − 08
*PSMD4* targets	7.36%	1.94%	1.50E − 04	0.04	10.69%	1.60%	2.70E − 11	9.71E − 06
Graft-versus-host disease	11.88%	3.35%	3.97E − 04	8.57E − 04	17.60%	2.72%	1.04E − 10	6.11E − 08
Allograft rejection	12.93%	3.38%	1.33E − 04	8.09E − 05	18.57%	3.02%	7.73E − 10	8.39E − 07
Antigen processing and presentation	5.64%	1.86%	2.43E − 07	0.04	9.27%	1.48%	3.98E − 10	9.40E − 05
Viral myocarditis	6.08%	1.61%	1.54E − 04	0.04	9.34%	1.47%	2.05E − 10	7.53E − 04

This table shows the significant pathways identified through the meta-analysis of cohort-specific pathway enrichment effects (random effects model) or tests (binomial tests). Results are shown for the meta-analysis of discovery cohorts alone, replication cohorts alone, and the discovery and replication cohorts combined. Gene set (Size) is the name of the gene set and the number of genes it included. Effect indicates the increased probability of a pathway component gene to have a significant measure of association (*U*-score ≤ 0.05) compared to a random gene. SE is the standard error of the effect; PAR-dr and WL-dr are the meta-analysis results for the paragraph and word list delayed recall assessments, respectively. RE_p is the *p*-value from the meta-analysis using the random effects model. Bin_p_A_ is the meta-analysis permutation-adjusted *p*-value from the binomial test. Bin_p is the meta-analysis exact *p*-value from the binomial test that does not adjust for multiple testing. ǂOnly six pathways were tested for replication, thus the exact path *p*-values (Bin_p) were used instead of the permutation-adjusted adjusted *p*-values (Bin_p_A_) when meta-analyzing pathway results across cohorts

The type I diabetes pathway association with WL-dr was replicated (*p*-value: Bin_*p* = 0.006) in independent cohorts. The PSMD4 targets exhibited marginal (*p*-value: RE_*p* = 0.046) replication for WL-dr. The meta-analytic effect sizes were small in the replication cohorts (− 1 ~ 3%). All six pathways met significance criteria (*p*-value: RE_*p* ≤ 4.86E-06 or Bin_p_A_ ≤ 0.05) for both delayed recall assessments in the joint meta-analysis of discovery and replication cohorts. However, the *p*-values and effect sizes (ranged from 6 to 19%) were generally attenuated compared to the values from the discovery cohorts alone.

#### Pathway enrichment of significant genes over cohorts (Approach 2)

The meta-analysis of gene associations across discovery cohorts yielded 69 and 173 genes significantly associated with PAR-dr and WL-dr, respectively (Table S5, *p*-value: Gene_*p* ≤ 1E-06); 66 genes were associated with both traits. Pathway enrichment analysis of significant genes identified the same six significant pathways (*p*-value: path_p_A_ ≤ 0.05; Table 3) as the meta-analysis of cohort-specific pathway enrichments (approach 1). Pathway effect sizes for PAR-dr (7 ~ 16%) were half those for WL-dr (13 ~ 31%). These six pathways harbored fifteen genes significantly associated with VDM in discovery cohorts (Table 4 and S6); eight and fifteen genes were significantly associated with PAR-dr and WL-dr, respectively. There were 75–100% of discovery cohorts showing the significant PAR-dr genes and 60–93% supporting the significant WL-dr genes (*U*-scores ≤ 0.05). All fifteen genes are members of the major histocompatibility complex (MHC), with eleven present in all six significant pathways. One gene, *HLA-DRA*, exhibited marginal evidence (*p*-value: Gene_*p* = 0.006) of replication for WL-dr with support from 38% of the replication cohorts.

**Table 3:** Significant pathways identified by Approach 2 (candidate gene enrichment analyses of summary gene associations from discovery cohorts)

	PAR-dr				WL-dr		
***Gene set***	***Size***	***Effect***	***SE***	***Path_p***_***E***_**ǂ**	***Effect***	***SE***	***Path_p***_***E***_** ǂ**
Type 1 diabetes	44	13.43%	0.69%	4.98E − 10	26.74%	1.10%	6.72E − 18
*PSMD4* targets	73	9.38%	0.53%	2.13E − 10	18.64%	0.85%	2.56E − 18
Graft-versus-host disease	42	14.08%	0.70%	3.71E − 10	28.04%	1.13%	3.56E − 18
Allograft rejection	38	15.58%	0.75%	1.97E − 10	31.04%	1.18%	8.87E − 19
Antigen processing and presentation	89	6.53%	0.49%	3.80E − 08	12.97%	0.77%	6.14E − 14
Viral myocarditis	73	8.01%	0.54%	1.14E − 08	15.90%	0.85%	5.13E − 15

This table displays pathways that were significantly enriched for memory-associated genes using Approach 2 and USGSA on the discovery cohorts. Candidate gene set enrichment analyses were run on 69 and 173 memory-associated genes for PAR-dr and WL-dr, respectively. Please refer to the legend of Table 1 for descriptions of Gene Set, Size, PAR-dr, and WL-dr. Effect is the increased probability of a memory-associated gene to be from the specified pathway versus a random gene. SE is the standard error of the effect estimate; Path_p_E_ǂ is the exact pathway *p*-value based on the hypergeometric distribution; Path_p_A_, the adjusted pathway *p*-value based on 10,000 permutations, took values “ < 0.001” for all displayed gene sets and outcomes and was omitted from the table

**Table 4:** Significant component genes from verbal declarative memory-associated pathways

	**Gene**	**GeneID**	**Meta discovery**		**Meta replication**		**Meta joint**	
			**PAR-dr (M/*Gene_p*)**	**WL-dr (M/*Gene_p*)**	**PAR-dr (M/*Gene_p*)**	**WL-dr (M/*Gene_p*)**	**PAR-dr (M/*Gene_p*)**	**WL-dr (M/*Gene_p*)**
MHC Class 1	*HLA-A*^a^	3105	5/1.54E − 05	10/2.32E − 10	1/0.34	1/0.34	6/8.09E − 05	11/3.76E − 09
	*HLA-B*^a^	3106	6/4.01E − 07	13/1.17E − 15	1/0.34	0/1.00	7/5.98E − 6	13/8.69E − 12
	*HLA-C*^a^	3107	8/3.91E − 11	14/8.73E − 18	0/1.00	0/1.00	8/3.50E − 7	14/3.25E − 13
	*HLA-E*^a^	3133	3/0.006	9/7.42E − 09	0/1.00	0/1.00	3/0.043	9/8.39E − 07
	*HLA-F*^a^	3134	3/0.006	9/7.42E − 09	2/0.057	0/1.00	5/8.57E − 04	9/8.39E − 07
	*HLA-G*^a^	3135	6/4.01E − 07	13/1.17E − 15	0/1.00	0/1.00	6/8.09E − 05	13/8.69E − 12
	*HLA-H*	3136	3/0.006	12/9.64E − 14	2/0.057	1/0.34	5/8.57E − 04	13/8.69E − 12
MHC Class II	*HLA-DMA*^a^	3108	6/4.01E − 07	11/5.53E − 12	0/1.00	1/0.34	6/8.09E − 05	12/1.96E − 10
	*HLA-DMB*^a^	3109	7/5.98E − 09	12/9.64E − 14	1/0.34	0/1.00	8/3.50E − 7	12/1.96E − 10
	*HLA-DPA1*^a^	3113	5/1.54E − 05	11/5.53E − 12	0/1.00	1/0.34	5/8.57E − 04	12/1.96E − 10
	*HLA-DPB1*^a^	3115	5/1.54E − 05	11/5.53E − 12	0/1.00	0/1.00	5/8.57E − 04	11/3.76E − 09
	*HLA-DPB2*	3116	7/5.98E − 09	11/5.53E − 12	0/1.00	0/1.00	7/5.98E − 06	11/3.76E − 09
	*HLA-DQA2*	3118	7/5.98E − 09	10/2.32E − 10	0/1.00	0/1.00	7/5.98E − 06	10/6.11E − 08
	*HLA-DQB2*	3120	7/5.98E − 09	11/5.53E − 12	0/1.00	0/1.00	7/5.98E − 06	11/3.76E − 09
	*HLA-DRA*^a^	3122	5/1.54E − 05	13/1.17E − 15	0/1.00	3/0.006	5/8.57E − 04	16/2.67E − 16

This table shows significant gene-based tests results for the component genes from the six memory-associated pathways. The binomial test was used to meta-analyze the gene associations (*U*-scores) across cohorts. Gene is the gene name or symbol; GeneID is the NCBI gene identifier; PAR-dr and WL-dr represent the results of the gene-based tests for the paragraph and word list delayed recall traits, respectively; M is the number of cohorts in which the gene had a *U*-score ≤ 0.05; Gene_p is the meta-analysis *p*-value (significant for values ≤ 1E − 06)

^a^Indicates a gene that is a component of all six memory-associated pathways

### DE analysis of significant pathway component genes

Fifteen significant genes from memory-associated pathways were differentially expressed by cognitive status in human brain tissue (Table 5); expression differed by Braak stage in astrocytes (*p* = 0.006) for the first data set (GDS4135) and by human immunodeficiency virus (HIV) cognitive impairment status (impaired infected versus uninfected controls) in brain tissues (*p* = 3.28E − 08) for the second data set (GDS4231). In basal ganglia of data set GDS4358, memory-associated pathway genes were differentially expressed across control, HIV-1 infected only (HIV-only), HIV-1 infected with substantial neurocognitive impairment (HIV-NCI), and HIV with neurocognitive impairment and HIV encephalitis (HIV-NCI-HIVE) groups (Trend I test; *p* = 3.33E − 05), as well as across the latter three groups after excluding control (Trend II test; *p* = 8.83E − 05). DE was found in the white matter tissue samples when controls were included (Trend I test; *p* = 0.03) but not when omitted (Trend II test; *p* = 0.50). No DE was found in the frontal cortex.

**Table 5:** Differential expression analysis of significant component genes from verbal declarative memory-associated pathways

**GEO_ID**	**PUBMED_ID**	**Organism**	**Tissue**	N_genes	**Contrast**	*p*-value
GDS4135	21705112	Human	Astrocytes	13	Braak stage I-II, III-IV, V-VI	0.006
GDS4231	21909266	Human	Brain tissues	13	HIV cognitive impairment vs uninfected control	3.28E − 08
GDS4358	23049970	Human	Basal ganglia	13	Trend I	3.33E − 05
					Trend II	8.83E − 05
			Frontal cortex	13	Trend I	0.74
					Trend II	0.99
			White matter	13	Trend I	0.03
					Trend II	0.50
GDS2082	15169854	House mouse	Hippocampus	12	15-month-old mice with age-related cognitive deficit vs 2-month-old normal mice	0.03
GDS2639	17376971	Norway rat	Hippocampus	6	Impaired vs. unimpaired cognition	0.016
GDS520	12736351	Norway rat	Hippocampus	5	Age 4, 14, and 24 months	0.015

This table contains results for the differential expression analysis (mean-rank test) of genes in memory-associated pathways by cognitive status in human and rodent samples. GEO_ID is the curated Gene Expression Omnibus data set identifier; PUBMED_ID is the PUBMED publication identifier. N_genes is the number of component genes that have expression measured in the given dataset (refer to Supplementary Tables S4 and S5 for the gene lists). Contrast indicates the cognitive function groups across which we contrasted the differential expression of component genes. *p*-value is the *p*-value from the gene-set differential analysis. The trend I test assessed differential expression across control, HIV-only, HIV-NCI, and HIV-NCI-HIVE statuses. The trend II test compared expression across the HIV-only, HIV-NCI, and HIV-NCI-HIVE statuses

We also examined the DE of homologous genes in three rodent studies of hippocampal tissue. Twelve and six homologous genes were available in the house mouse and Norway rat, respectively (Table S7). Mean-rank tests confirmed DE of these genes in the hippocampus of house mice with age-related spatial memory deficits compared to young mice (*p* = 0.03) for the data set GDS2082, Norway rats with impaired versus normal cognition (*p* = 0.016) for the data set GDS2639, and Norway rats with age-dependent cognitive decline at 4, 14, and 24 months for the data set GDS520 (*p* = 0.015).

## Discussion

Debette et al. conducted meta-analyses of PAR-dr and WL-dr GWAS data across cohorts participating in the CHARGE consortium. They identified a significant VDM-associated variant located near genes involved in the immune response and found a correlation between memory risk variants and gene expression in human hippocampal cells. They also conducted pathway analyses focused on molecules with physical contact [2]. In this study, we expanded beyond the confines of prior research and adopted a more comprehensive approach to investigate the potential functions of VDM-associated variants. Our investigation demonstrated that VDM-associated variants are in high linkage disequilibrium with eQTLs across all 44 tissues and meQTLs in the hippocampus. Our analyses indicated that VDM-associated genes have reduced odds of being highly expressed in four specific brain tissues. Furthermore, VDM-associated genes appeared to be regulated by thirty-one TFs and two microRNAs, while also being implicated in immune function. Our analyses highlighted six pathways, including one relevant to type I diabetes, significantly correlated with both PAR-dr and WL-dr. Remarkably, these pathways encompassed fifteen MHC genes intricately tied to VDM performance. These MHC genes exhibited differential expression by cognitive status in brain tissues.

This investigation showcased the ability of multi-omics and pathway analyses to attribute function to GWAS associations. Our findings implicate gene expression regulation and immunity as functions underlying VDM genetic associations in older non-demented individuals of European descent. The multi-omics analyses showed that PAR-dr and WL-dr single-variant associations exhibited LD with eQTLs in every tissue and meQTLs in the hippocampus, bolstering evidence that trait-associated variants are enriched in eQTLs [9, 33] and regions involved in expression regulation [34, 35]. The connection between VDM-associated variants and meQTLs in hippocampal tissue echoed the association of Alzheimer’s neuropathology and disease with methylation changes in brain tissue (including the hippocampus) [36–38]. We observed a lack of tissue specificity in the eQTL analysis which is similar to other memory-related traits [39]. However, the strongest eQTL relationship was with PAR-dr genetic associations in the hippocampus, a brain region involved in the acquisition of new memories and verbal and narrative memory [40].

Stronger WL-dr gene associations were connected to expression downregulation in four brain tissues (the anterior cingulate cortex, caudate, hippocampus, and pituitary gland), while stronger PAR-dr and/or WL-dr gene associations implicated regulation by thirty-one TFs and two microRNAs and classification as immune genes. Sequence variation in TFs and their binding site clusters, as well as microRNA expression levels (specifically hsa-miR-218–1-5p), have been associated with AD [41–43]. Similarly, the increased odds of immune function ascribed to genetic associations are supported by previous studies of AD [41, 44].

While Debette et al. utilized summarized statistics to pinpoint VDM-associated pathways through a network of molecules with physical interactions [2], our study took a different approach. We leveraged the Molecular Signatures Database (MSigDB) to broaden our pathway analysis to include 10,295 curated gene sets. In this endeavor, we gathered individual GWAS results from each cohort and examined VDM-associated pathways within their respective contexts. We conducted meta-analysis using the random-effect model to gauge pathway enrichment effects across cohorts and employed binomial meta-analysis to assess if VDM-associated pathways within cohorts exhibited non-random trends. To validate our findings, replication cohorts were examined alongside the original discovery cohorts in this study.

The pathway enrichment analysis identified six VDM-associated pathways (type 1 diabetes, graft-versus-host disease, allograft rejection, antigen processing and presentation, viral myocarditis, and targets of PSMD4 regulation) which were all interrelated within the framework of immunity. Antigen presentation, the process by which MHC proteins bind and transport ingested antigens to the surface of antigen presenting cells where they can be recognized by T-cells [45, 46], is critically involved in the early stages of type 1 diabetes (during the autoimmune destruction of pancreatic beta cells [47]), graft-versus-host disease (when T-cells from a foreign donor graft attack antigens expressed by the recipient [48]), allograft rejection (when T-cells from the recipient directly or indirectly attack antigens from transplanted tissue from a genetically non-identical human donor [49]), viral myocarditis (when viral antigens are presented to T-cells following an infection of cardiac myocytes [50]), and the induction of inflammatory cytokine production (several cytokines are members of the PSMD4 targets pathway [51, 52]).

The type I diabetes pathway association was replicated in independent cohorts and is biologically plausible. Insulin deficiency may reduce VDM performance through altered cerebral glucose metabolism, neurotransmitter expression/activity, neurotrophins, long-term potentiation, or inflammatory responses [47]. Increasing plasma insulin levels intravenously while preserving euglycemia aids VDM (story and word list recall) in both healthy adults and AD patients [47]. Similarly, acute and chronic intranasal insulin administration improved verbal memory in AD patients and healthy young adults, respectively [47]. In general, adults with type I diabetes perform worse on memory tests than non-diabetics [53]. AD patients (who often exhibit reduced VDM performance) have decreased hippocampal glucose consumption, hippocampal insulin receptor mRNA, and brain insulin receptor protein levels compared to age-matched controls [54, 55]. Gene expression studies also link diabetes with the AD pathway [56].

Our pathway enrichment findings may reflect a single pathway or MHC gene associations. The six VDM-associated pathways shared eleven MHC genes and collectively harbored fifteen MHC genes exhibiting differential expression by cognitive status in human and rodent brain tissues. MHC I proteins may be required for hippocampus-dependent memory [57]. An MHC II gene (*HLA-DRB1*) was associated with delayed verbal recall performance in older non-demented individuals [58] and AD [59], while hippocampal MHC II protein levels were inversely associated with mini-mental state examination scores [60]. Several MHC genes associated with VDM in this investigation (including the marginally replicated *HLA-DRA*) have been associated with AD (*HLA-A, HLA-B, HLA-DRA* [61–63]) or showed increased hippocampal (*HLA-DMA, HLA-DMB, HLA-DPA1, HLA-DRA* [60]) or pre-frontal cortex (*HLA-A, HLA-C, HLA-E, HLA-F, HLA-G, HLA-DPB*1 [60]) expression in mild AD dementia cases compared to non-demented controls. MHC genes may influence memory through their effects on synaptic plasticity, development, morphology, and function [57, 64–66].

This investigation had a few limitations, including the lack of stringent replication for the multi-omics and pathway analyses. The replication cohorts were mainly restricted to women with some college education and had different PAR-dr and WL-dr assessments compared to the discovery cohorts. Each cohort-specific GWAS used HapMap II CEU-imputed data, which has a sparser gene coverage than 1000 Genomes-imputed or whole genome/exome sequence data. Therefore, the findings may be less accurate due to the omission of rare genetic variation of large effect. The original cohort-specific findings assumed additive genetic effects, thus we possibly missed genes and pathways containing dominant or recessive variant effects.

In this research, we investigated the relationship between VDM-associated variants and eQTLs and meQTLs. Specifically, we leveraged logistic regression to evaluate the linear association between the negative logarithm of *p*-values and the logarithm of odds that variants are in high LD with eQTLs and meQTLs. However, one limitation is that our research cannot definitively establish whether the same variant is causally linked to VDM and the regulation of eQTLs and meQTLs. Therefore, it is worthwhile to explore the identification of VDM variants that may be responsible for both the GWAS signals and regulatory effects by employing techniques such as colocalization and fine-mapping approaches [67, 68]. Additionally, we selected a threshold of *r*^2^ = 0.8 to determine if a genetic variant is in high LD with eQTLs and meQTLs. However, selection using a different threshold may impact the findings, thus incorporating more sophisticated methods such as LD scoring may enhance the robustness of our tests.

Our study may also be hindered by different gene association measures, selection of gene boundaries for SNP mapping, the incompleteness of omics databases, and annotation biases [5]. Lastly, this investigation included participants of European ancestry, thus findings may not generalize to other racial or ethnic groups.

## Conclusions

In conclusion, our results add to the mounting evidence implicating expression regulation, immunity, and insulin deficiency in memory impairment. Future studies should attempt to dissect the molecular mechanisms underlying these relationships, so treatments can be developed to combat the increasing burden of cognitive decline and AD on society.

### Supplementary Information

**Additional file 1: Supplemental Text. Table S1.** Sample size and the number of SNPs in the paragraph delayed recall GWAS from each discovery and replication cohort. **Table S2.** Sample size and the number of SNPs in the word list delayed recall GWAS from each discovery and replication cohort. **Table S3.** Tissue-specific relationships between delayed recall test (PAR-dr and WL-dr) summary SNP associations and eQTLs and meQTLs. **Table S4.** Relationship Between Delayed Recall Summary Gene Associations and Transcription Factor Genes. **Table S5.** Significant Genes Associated with Paragraph Delayed Recall (PAR-dr) and Word List Delayed Recall (WL-dr). **Table S6.** Significant component genes in the six memory-associated pathways. **Table S7.** Homologous genes in memory-associated pathways for differential expression analysis. **Figure S1.** GWAS cohorts and microarray expression datasets. **Figure S2.** Design of the pathway analyses. **Figure S3.** Forest plots of significant pathway enrichment effects and *p*-values from discovery cohorts (Approach 1).

## Data Availability

The datasets used and analyzed during the current study are available from the corresponding author on request.
