# Supplementary material for: Multi-omics and pathway analyses of genome-wide associations implicate regulation and immunity in verbal declarative memory performance
Source: Alzheimers Res Ther. 2024 Jan 20;16:14. doi: 10.1186/s13195-023-01376-6 (PMC10799499; doi:10.1186/s13195-023-01376-6)
Supplement: Supplementary file 1 — Additional file 1: Supplemental Text. Table S1. Sample size and the number of SNPs in the paragraph delayed recall GWAS from each discovery and replication cohort. Table S2. Sample size and the number of SNPs in the word list delayed recall GWAS from each discovery and replication cohort. Table S3. Tissue-specific relationships between delayed recall test (PAR-dr and WL-dr) summary SNP associations and eQTLs and meQTLs. Table S4. Relationship Between Delayed Recall Summary Gene Associations and Transcription Factor Genes. Table S5. Significant Genes Associated with Paragraph Delayed Recall (PAR-dr) and Word List Delayed Recall (WL-dr). Table S6. Significant component genes in the six memory-associated pathways. Table S7. Homologous genes in memory-associated pathways for differential expression analysis. Figure S1. GWAS cohorts and microarray expression datasets. Figure S2. Design of the pathway analyses. Figure S3. Forest plots of significant pathway enrichment effects and p-values from discovery cohorts (Approach 1). [file 13195_2023_1376_MOESM1_ESM.pdf]

1. Cohorts

Discovery Studies with Memory GWAS

The CHARGE consortium includes large prospective community-based cohort studies with genome-wide variation data and multiple phenotypes involving measures of memory functions[1]. These cohorts and several other community-based cohorts have collaborated to complete the genome-wide association study (GWAS) of memory[2]. All of the discovery cohorts in the GWAS were analyzed for memory-related genetic pathways and some details are described as follows.

The Atherosclerosis Risk in Communities Study (ARIC)

The ARIC study is a prospective population-based study of atherosclerosis and clinical atherosclerotic diseases in 15,792 men and women, including 11,478 white participants, drawn from 4 United States communities (Suburban Minneapolis, Minnesota; Washington County, Maryland; Forsyth County, North Carolina; and Jackson, Mississippi). In the first 3 communities, the sample reflects the demographic composition of the community. In Jackson, only black residents were enrolled. Participants were between age 45 and 64 years at their baseline examination in 1987-1989 when blood was drawn for DNA extraction and participants consented to genetic testing [3]. A total of 15,020 participants, of which 10,898 were white, were genotyped at the Broad Institute, Boston, Massachusetts, and 9,345 of the latter passed QC criteria for genotyping and were available for analysis after application of all exclusion criteria. Vascular risk factors and outcomes, including transient ischemic attack and stroke, were determined in a standard fashion[4]. The second clinical examination of the ARIC Study cohort in 1990–1992 included a neuropsychological evaluation[5], with the Delayed Word Recall Test (DWRT)[6], to assess memory performance. Given the relatively young age of the cohort at the second examination (age range = 48-67 years) and low expected prevalence of dementia, a formal assessment for dementia was not performed. Among white participants with genome-wide data, after excluding 275 participants with a history of stroke, 9,188 participants were available for a GWAS of delayed word list recall (visually presented word list). In addition, among white participants with genome-wide data, 509 have recently completed a detailed neuropsychological test battery as part of an ancillary study[7,8], including the logical memory test from the revised version of the Wechsler Memory Scale (WMS-R). After excluding 79 participants with a history of stroke, 430 ARIC white participants were available for a GWAS of delayed paragraph recall.

The Cardiovascular Health Study (CHS)

The CHS is a population-based cohort study of risk factors for vascular disease in adults 65 years or older conducted across 4 field centers in the United States: Sacramento County, California; Washington County, Maryland; Forsyth County, North Carolina; and Pittsburgh, Allegheny County, Pennsylvania[9]. The original predominantly white cohort of 5,201 persons was recruited in 1989-1990 from a random sample of people on Medicare eligibility lists. An additional 687 African-Americans were enrolled in 1992-1993, for a total sample of 5,888. Vascular risk factors and outcomes, including transient ischemic attack, stroke and dementia, were determined in a standard fashion[10,11]. DNA was extracted from blood samples drawn on all participants who consented to genetic testing at their baseline examination in 1989-90 or 1992-1993. In 2007-2008, genotyping was performed at the General Clinical Research Center's Phenotyping/Genotyping Laboratory at Cedars-Sinai on 3,980 CHS participants who were free of cardiovascular disease at baseline and who had DNA available for genotyping. Because most other cohorts were predominantly white, the African

American participants were excluded from this analysis to limit the potential for false positive associations due to population stratification. Among white participants, genotyping was attempted in 3,397 participants and was successful in 3,295 persons. As part of the CHS Cognition Study[11,12], in 1992-94 and again, in 1997-99, participants were invited to undergo a detailed neuropsychological assessment including the CVLT[13] and the logical memory test from the WMS-R [14]. Among participants with genome-wide data: after exclusion of 47 participants with dementia and of 26 participants with history of stroke, 334 participants were available a GWAS of delayed word list recall (CVLT); after exclusion of 4 participants with dementia, 472 participants were available for a GWAS of delayed paragraph recall.

Framingham Heart Study (FHS)

The FHS is a three-generation, single-site, community-based, prospective cohort study that was initiated in 1948 to investigate risk factors for cardiovascular disease including stroke. It now comprises 3 generations of participants: the original cohort followed since 1948 (Original)[15]; their offspring and spouses of the offspring, followed since 1971 (Offspring)[16]; and children from the largest offspring families enrolled in 2000 (Gen 3)[17]. The Original cohort enrolled 5,209 men and women who comprised two-thirds of the adult population then residing in Framingham, MA, USA. Survivors continue to receive biennial examinations. The Offspring cohort comprises 5,124 persons (including 3,514 biological offspring) who have been examined approximately once every 4 years. Participants in the first two generations were invited to undergo an initial neuropsychological test battery in 1999-2005[18], including the logical memory test from the Original WMS[19]. Neuropsychological testing in Gen 3 only began in 2009 and is not included in these analyses. The population of Framingham was virtually entirely whites in 1948 when the Original cohort was recruited. Vascular risk factors and outcomes, including transient ischemic attack, stroke and dementia, were identified prospectively since 1948 through an ongoing system of FHS clinic and local hospital surveillance[20,21]. Participants had DNA extracted and provided consent for genotyping in the 1990s. Genotyping was performed at Affymetrix (Santa Clara, CA) through an NHLBI funded SNP-Health Association Resource (SHARe) project and was successful in 4,519 persons from the Original and Offspring cohorts. Of these 4,519 persons, 4,116 were alive in 1999 when the neuropsychological study began. Of these, 2,642 participants have undergone neuropsychological testing including logical memory. We excluded 30 participants with a neurological condition that might confound the cognitive assessment (e.g. brain tumor or severe head injury), 7 participants with dementia and 52 participants with a history of stroke. Of the remaining 2,553 participants, 2,493 were aged 45 years or older and were available for a GWAS of delayed paragraph recall.

Lothian Birth Cohort 1921 (LBC1921) and 1936 (LBC1936)

These samples include surviving participants from the Scottish Mental Surveys of 1932 or 1947 (SMS1932 and SMS1947), having been born, respectively in 1921 (LBC1921) and 1936 (LBC1936)[22-24]. They were all Caucasian and almost all lived independently in the Lothian region (Edinburgh city and surrounding area) of Scotland. The LBC1921 cohort comprised 550 members while the LBC1936 cohort included 1,091 participants. At age 79 approximately, LBC1921 participants underwent a neuropsychological examination including the logical memory test from the WMS-R[14]. At age 70, LBC1936 participants took a battery of cognitive tests[22], including the logical memory test from the Wechsler Memory Scale-IIIUK (WMS-IIIUK)[25]. Genotyping was performed at the Wellcome Trust Clinical Research Facility (WTCRF) Genetics Core, Western General Hospital, Edinburgh. Among participants with good quality genome-wide data, after exclusion of 5 participants for dementia and 43 participants for history of stroke, 469 individuals from the LBC1921 cohort were available for a GWAS on paragraph delayed recall . Among participants with genome-wide data, after

exclusion of 50 participants for history of stroke, 953 individuals from the LBC1936 cohort were available for a GWAS on delayed paragraph recall.

Rush Memory and Aging Project (MAP)

The MAP, started in 1997, enrolled older men and women from assisted living facilities in the Chicago area with no evidence on dementia at baseline[26]. Since October 1997, 1,285 participants completed their baseline evaluation, of whom 1,118 were non-Hispanic white. The study was approved by the institutional review board of Rush University Medical Center. The follow-up rate of survivors exceeds 90%. Similar to the ROS, participants agreed to annual clinical evaluations and signed both an informed consent and an Anatomic Gift Act form donating their brains, spinal cords, and selected nerves and muscles to Rush investigators at the time of death[27,28]. A more detailed description of the MAP has been published previously[27,28]. Participants were invited to take a neuropsychological test battery, including delayed recall of Story A from the logical memory subset of the Wechsler Memory Scale-Revised[14], and delayed word list recall from the CERAD battery[29]. DNA was extracted from whole blood, lymphocytes, or frozen postmortem brain tissue. Genotyping was performed at the Broad Institute's Center for Genotyping and the Translational Genomics Research Institute. Among participants with available memory tests and genome-wide genotypes, after exclusion of 51 participants with dementia and 86 participants with a history of stroke, 751 individuals were available for a GWAS of delayed paragraph recall and delayed word list recall (visually presented word list).

Orkney Complex Disease Study (ORCADES)

ORCADES is an ongoing, family-based, cross-sectional study that seeks to identify genetic factors influencing cardiovascular and other disease risk in the population isolate of the Orkney Isles in northern Scotland[30]. The North Isles of Orkney, the focus of this study, consist of a subgroup of ten inhabited islands with census populations varying from ~30 to ~600 people on each island. The first phase of data collection was carried out in Orkney between 2005 and 2007. Informed consent and blood samples were provided by 1019 Orcadian volunteers who had at least one grandparent from the North Isles of Orkney. Participants were invited to take a neuropsychological test battery including the logical memory test adapted from the Original Wechsler Memory Scale[19]. Genotyping was performed at the Helmholtz Centre in Munich on a subset of 719 participants. An additional 169 individuals were genotyped by Integragen in Paris. Among participants with genome-wide data, we excluded 7 participants with a history of stroke. Of the 537 remaining individuals, aged 20 years or older, 419 participants aged 45 years or older were available for a GWAS of delayed paragraph recall.

Religious Orders Study (ROS)

The ROS, started in 1994, enrolled Catholic priests, nuns, and brothers, aged 53 years from about 40 groups in 12 states[26]. Since January 1994, 1,132 participants completed their baseline evaluation, of whom 1,001 were non-Hispanic white. The study was approved by the institutional review board of Rush University Medical Center. The follow-up rate of survivors exceeds 90%. Participants were free of known dementia at enrollment, agreed to annual clinical evaluations, and signed both an informed consent and an Anatomic Gift Act form donating their brains at time of death[27]. A more detailed description of the ROS has been published previously[27]. Participants were invited to take a neuropsychological test battery, including delayed recall of Story A from the logical memory subset of the Wechsler Memory Scale-Revised[14], and delayed word list recall from the Consortium to Establish a Registry for Alzheimer's Disease (CERAD) battery[29]. DNA was extracted from whole blood, lymphocytes, or frozen postmortem brain tissue. Genotyping was performed at

the Broad Institute's Center for Genotyping and the Translational Genomics Research Institute. Among participants with available memory tests and genome-wide genotypes, after exclusion of 58 participants with dementia and 65 participants with a history of stroke, 687 individuals were available for a GWAS of delayed paragraph recall and delayed word list recall (visually presented word list).

Aging Gene-Environment Susceptibility - Reykjavik Study (AGES-Reykjavik)

The AGES-Reykjavik Study is a single center prospective cohort study based on the Reykjavik Study. The Reykjavik Study was initiated in 1967 by the Icelandic Heart Association to study cardiovascular disease and risk factors. The cohort included men and women born between 1907 and 1935 who lived in Reykjavik at the 1967 baseline examination. Re-examination of surviving members of the cohort was initiated in 2002 as part of the AGES-Reykjavik Study. The AGES-Reykjavik Study is designed to investigate aging using a multifaceted comprehensive approach that includes detailed measures of brain function and structure. All cohort members were European Caucasians. Briefly, as part of a comprehensive examination, all participants answered a questionnaire, underwent a clinical examination and had blood drawn[31]. All consenting participants were offered to take a neuropsychological test battery[32], including the California Verbal Learning Test (CVLT)[13], to assess memory performance. Among participants with genome-wide data, after exclusion of 126 participants with dementia and 266 participants with a history of stroke, 2616 participants were available for a GWAS of delayed word list recall (CVLT).

Erasmus Rucphen Family (ERF)

The Erasmus Rucphen Family (ERF) study is a family-based cohort study in a genetically isolated population in the Netherlands[33,34], including 3,000 participants. Participants are all descendants of a limited number of founders living in the 19th century. Extensive genealogical data is available for this population. The study protocol included venous puncture for DNA isolation and chemistry, cognitive evaluation, cardiovascular examination, eye assessments and body composition measurements. All participants gave informed consent and the study was approved by the medical ethics committee at Erasmus MC University Medical Center. Genotyping was done at the Human Genotyping Facility, Genetic Laboratory Department of Internal Medicine, Erasmus MC, Rotterdam, and at the Genotyping Center of Leiden University, The Netherlands. In total, 2,385 samples from the ERF Study were available with good quality genotyping data. Participants were invited to undergo a neuropsychological evaluation[35], which included the Dutch version of Rey's Auditory Verbal Learning Test (RAVLT)[36]. Among participants with genome-wide data, we excluded 20 individuals with a history of stroke. Of the remaining 2 119 participants, aged 20 years or older, 1,267 participants were aged 45 years or more and were available for performance GWAS of delayed word list recall (RAVLT).

Genetic Epidemiology Network of Arteriopathy (GENOA)

GENOA is a study of hypertensive sibships designed to investigate the genetic underpinnings of hypertension and target organ damage. In the initial phase of the GENOA study (Phase I: 1996-2001), all members of sibships containing $\geqslant 2$ individuals with essential hypertension clinically diagnosed before age 60 were invited to participate, including both hypertensive and normotensive siblings (1,583 non-Hispanic whites from Rochester, MN, and 1,841 African Americans from Jackson, MS). The diagnosis of essential hypertension was established based on blood pressure levels measured at the study visit (>140 mmHg average systolic BP or >90 mmHg average diastolic BP) or a prior diagnosis of hypertension and current treatment with antihypertensive medications. Exclusion criteria were secondary hypertension, alcoholism or drug abuse, pregnancy, insulin-dependent diabetes mellitus, or active malignancy. In the second phase of

the GENOA study (Phase II: 2000-2004), 1,241 white and 1,482 African American participants were successfully re-recruited to measure potential target organ damage due to hypertension. The Genetics of Microangiopathic Brain Injury (GMBI) study (2001-2006) is an ancillary study of GENOA undertaken to investigate susceptibility genes for ischemic brain injury. Phase II GENOA participants that had a sibling willing and eligible to participate in the GMBI study underwent a neurocognitive testing battery to assess several domains of cognitive function including Rey's Auditory Verbal Learning Test (RAVLT)[36] (967 whites and 1,010 African Americans). Genotyping was performed at the Mayo Clinic, Rochester (MN). GENOA white participants who were less than 45 years of age (N=56) or had history of stroke (N=22) were excluded from the analysis, leaving a total of 889 participants. Among participants with genome-wide genotype data, 758 GENOA white participants from 378 sibships were available for a GWAS on delayed word list recall (RAVLT).

Helsinki Birth Cohort Study (HBCS)

The source cohort for the HBCS comprised 4,130 women and 4,630 men born as singletons at Helsinki University Central Hospital during 1934-44, who had birth and child welfare records and were living in Finland in 1971[37]. To approach an intended sample size of N=2,000, a random subsample of 2,902 subjects was invited to participate in the study; 2,003 of them (1,075 women and 928 men) were finally included[38]. Participants who could come to the examination center were invited to take a neuropsychological test battery, including the delayed word list recall from the CERAD battery[29]. 1,063 participants attended neuropsychological testing between February 2005 and February 2011. DNA was extracted from 1,728 randomly selected participants of the HBCS. Genotyping was conducted at the Wellcome Trust Sanger Institute, Cambridge, UK. Among participants with available cognitive tests and genome-wide genotypes, after exclusion of 19 participants with a history of stroke, 888 individuals were available for a GWAS of delayed word list recall (visually presented word list). The study was approved by the Institutional Review Board of the National Public Health Institute, and informed consent was obtained from all participants.

Croatian Cohorts: Split and Korčula

The Croatia-Korčula study is part of a larger genetic epidemiology research program in Croatian island isolates, "10,001 Dalmatians." The genetic epidemiology research program in Croatian island isolates began in 1999[39], then expanded to study human genetic variation and effects of isolation and inbreeding[40,41], and finally entered the phase of focusing on diseases and gene mapping studies[42-44]. A total of 969 participants were included in the CROATIA-Korčula study. The Croatia-Split study included 535 persons collected in 2009 from the general (outbred) population of Split. Split has a population of >100,000 and is the second largest city in Croatia. Participants from the Croatia-Korčula and Croatia-Split studies were invited to undergo a neuropsychological examination including the Rey's Auditory Verbal Learning Test (RAVLT)[36]. Croatia- Korčula genotyping was performed at the Institute of Human Genetics, Helmholtz Zentrum München, Germany and CROATIA-Split genotyping was performed at AROS Applied Biotechnology, Aarhus, Denmark. Genotyping was successful in 898 and 499 participants, respectively, for Croatia-Korčula and Croatia-Split. Among participants with genome-wide data, 25 and 10 participants with a history of stroke were excluded from the Croatia-Korčula and Croatia-Split studies, respectively. Of the remaining 577 and 471 individuals, aged 20 years or more, 472 and 303 participants aged 45 years or older were available for a GWAS of delayed word list recall (RAVLT).

Rotterdam Study

The Rotterdam Study is a population-based prospective cohort study among inhabitants of a district of Rotterdam (Ommoord), The Netherlands, and aims to examine the determinants of disease and health in the elderly with a focus on neurogeriatric, cardiovascular, bone, and eye disease[45 46,47]. In 1990-1993, 7,983 persons 55 years of age or over (Rotterdam Study-I) participated and were re-examined every 3 to 4 years. In 1999, 3,011 individuals who had become 55 years of age or moved into the study district since the start of the study were added to the cohort (Rotterdam Study-II), and in 2006 a further extension of the cohort was initiated in which 3,932 subjects aged 45–54 years and living in the same district were included (Rotterdam Study-III)[48]. All participants had DNA extracted at their first visit. Genotyping was attempted in participants with high-quality extracted DNA. Genotyping was done at the Human Genotyping Facility, Genetic Laboratory Department of Internal Medicine, Erasmus MC, Rotterdam, the Netherlands. Participants underwent several neuropsychological tests at the baseline and follow-up examinations[49], including a 15-word verbal learning test based on Rey's recall of words[50]. Participants are continuously monitored for major events, including dementia and stroke, by automated linkage of the general practitioners' records and hospital discharge files with the study database[51,52]. Among participants with good quality genome-wide data: after exclusion of participants with dementia (N=124 for the Rotterdam Study-I, N=56 for the Rotterdam Study-II and N=6 for the Rotterdam Study-III) and participants with a history of stroke (N=168 for the Rotterdam Study-I and N=102 for the Rotterdam Study-II), 2,067 participants from the Rotterdam Study-I, 1,533 participants from the Rotterdam Study-II and 1,935 participants from the Rotterdam Study-3 were available for a GWAS of word list delayed recall (visually presented word list).

Study of Health in Pomerania (SHIP)

The Study of Health in Pomerania is a population-based epidemiological study in the region of Western Pomerania, Germany[53]. In brief, from the total population of West Pomerania comprising 213,057 inhabitants in 1996, a two-stage stratified cluster sample of adults aged 20–79 years was drawn. The net sample (without migrated or deceased persons) comprised 6,265 eligible subjects, out of which 4,308 completed their baseline examinations. From July 2007 to October 2010 the Life-Events and Gene-Environment Interaction in Depression (LEGENDE) study was carried out in the SHIP cohort. A diagnostic interview for mental disorders was performed based on Diagnostic and Statistical Manual for Mental Disorders (IV edition) diagnostic criteria[54]. As part of the SHIP-LEGENDE project[55], participants have been invited to take a cognitive test battery, including a German adaptation of Rey's Auditory Verbal Learning Test (RAVLT). Genotyping was performed at Affymetrix (Santa Clara, CA). The genetic data analysis workflow was created using the Software InforSense. Genetic data were stored using the database Caché (InterSystems). Among participants with available cognitive tests and genome-wide genotypes, we excluded 62 participants with dementia and 19 participants with a history of stroke. Of the remaining 2,027 individuals, aged 20 years or older, 1,474 participants aged 45 years or older were available for a GWAS of delayed word list recall (RAVLT).

Tasmanian Study of Cognition and Gait (TASCOG)

TASCOG is a study of cerebrovascular mechanisms underlying gait, balance and cognition in a population-based sample of Tasmanian people aged at least 60 years. Individuals aged 60–86 years (N=395) living in Southern Tasmania, Australia, were randomly selected from the electoral roll to participate in the study. Individuals were excluded if they lived in a nursing home, had a contraindication for magnetic resonance scanning (MRI) or were unable to walk without a gait aid[56]. Participants were invited to take a

neuropsychological test battery[57], including among other tests the Hopkins Verbal Learning Test (HVLT)[58]. DNA was extracted from peripheral blood samples by proteinase K digestion following cell lysis, then phenol-chloroform purification. DNA was genotyped at the Diamantina Institute and Institute of Molecular Biosciences, University of Queensland, Australia, for 370 participants, and call rates were greater than 97% for all samples. Genotypes for 22 individuals were excluded, either because they were closely related to other individuals, they were outliers in a population ancestry analysis or their sex predicted from genotypes did not match sex as recorded in the database. Among the 348 remaining participants with available genome-wide data, after exclusion of 2 participants with dementia and 15 participants with a history of stroke, 331 individuals were available for performance GWAS of delayed word list recall (HVLT).

Replication Studies with Memory GWAS

Three cohorts in the replication study of the GWAS analysis[2] have also completed the whole-genome association analysis and been investigated as replication studies of memory-related genetic pathways. These cohorts were described as follows.

Sydney Memory and Ageing Study (Sydney MAS)

The Sydney MAS was initiated in 2005 to examine the clinical characteristics and prevalence of mild cognitive impairment and related syndromes, and to determine the rate of change in cognitive function over time[59]. Participants were invited to take a neuropsychological test battery, including among other tests the RAVLT[36], and Story A of the Wechsler Memory Scale, 3rd edition, (WMS-III)[60]. DNA was genotyped at the Ramaciotti Centre, UNSW, Australia. Among the 925 participants with available genome-wide data, after exclusion of 37 participants with a history of stroke, 880 individuals were available for performing a GWAS of delayed word list recall (RAVLT) and 887 for delayed paragraph recall (WMS-III).

Women's Genome Health Study (WGHS)

The WGHS is a large cohort for genome-wide genetic analysis of a wide range of clinical phenotypes among >25,000 women, 45 years or older at baseline and with ongoing follow-up observation, now for approximately 18 years[61]. The population is derived from participants in the Women's Health Study who provided a blood sample at baseline. By design, participants included in the WGHS were free from dementia and stroke at baseline. Women above 70 years of age were contacted for a baseline telephone cognitive assessment, including a measure of delayed paragraph recall using the East Boston Memory Test, and the Telephone Interview of Cognitive Status 10–item word list. Genome-wide genotyping in the WGHS was performed using the Illumina (San Diego, CA) HumanHap 300 Duo "+" platform including a total 339,596 SNPs passing quality control filters among 23,294 participants with verified European ancestry. Among participants with available memory tests and genome-wide genotypes, 3,542 individuals of verified European ancestry were available for genetic analysis of delayed word list recall (TICS) and of delayed paragraph recall.

Nurses' Health Study (NHS)

The NHS began in 1976, when 121,700 female registered nurse women, aged 30 to 55 years, living in 11 USA states completed a mailed questionnaire on lifestyle and health. Every 2 years, follow-up questionnaires have been mailed to participants to update their information, and >90% follow-up of the total possible person-time has been maintained[62]. For the study of cognitive function, participants aged 70 years and older free of diagnosed stroke were selected (dementia diagnosis was not ascertained). From 1995 to 2001, 21,085 eligible women were contacted for a baseline telephone cognitive assessment, including a measure of

delayed paragraph recall using the East Boston Memory Test, and the Telephone Interview of Cognitive Status 10 –item word list; 19,415 (92%) completed the interview. The study was approved by the Institutional Review Board of the Brigham and Women's Hospital[62]. Genotyping was conducted at the Broad Institute using a nested case-control design. Among participants from 6 independent case-control studies in the NHS cohort with available memory tests and genome-wide genotypes, 2,066 / 1,773 individuals were available for a GWAS of delayed paragraph / word list recall (NHS-CGEM): 756 / 655 from the diabetes case-control dataset (NHS-T2D), 358 / 307 from the coronary heart disease case-control dataset, 553 / 450 from the breast cancer case-control dataset (NHS-CHD), 179 / 166 from the glaucoma case-control dataset (NHS-GLAU), 80 / 76 from the kidney stone case-control dataset (NHS-KS), and 141 / 119 from the endometrial cancer case-control dataset (NHS-ENDO).

2. Samples for the Differential Gene Expression Analyses

We performed a gene-set differential expression (DE) analysis using significant component genes (meta-analysis gene p-value, Gene_p ≤ 1E-06) from verbal declarative memory-associated pathways. Our aim was to examine if these pathway genes collectively had stronger DE by cognitive status in brain tissues compared to an identical number of random genes. Three curated human gene expression studies of cognitive traits were selected from the Gene Expression Omnibus [63]. The first study (ID: GDS4135) tested DE over Braak stages I-II, III-IV, and V-VI of neurofibrillary tangle pathology in astrocytes [64]. The second study (ID: GDS4231) examined DE between HIV-1-associated neurocognitive disorders and uninfected controls in postmortem brain tissue [65]. The third study (ID: GDS4358) investigated the trend of expression changes across uninfected (controls), HIV-1 infected only (HIV-only), HIV-1 infected with substantial neurocognitive impairment (HIV-NCI), and HIV-NCI with HIV encephalitis (HIV-NCI-HIVE) participants in tissue samples from basal ganglia, the frontal cortex, and white matter [66]. For the third study, we performed tests both including (Trend I test) and omitting (Trend II test) the uninfected controls.

Three rodent DE studies were performed using homologues (identified using the NCBI HomoloGene tool [67]) of the significant memory-associated pathway genes in hippocampal tissue. The first study (ID: GDS2082) investigated DE in 15-month-old house mice with age-related spatial memory deficits versus 2-month-old normal mice [68]. The second study (ID: GDS2639) compared expression in Norway rats by cognitive impairment status (impaired versus unimpaired cognition) [69]. The third study (ID:

GDS520) investigated DE among Norway rates with age-dependent cognitive decline over 4, 14, and 24 months [70].

3. Supplemental Tables

Table S1: Sample size and the number of SNPs in the paragraph delayed recall GWAS from each discovery and replication cohort

Discovery Study	Size	Mapped Genes	SNPs Mapped to Genes	Independent SNPs Mapped to Genes
ARIC	430	36,370	1,629,523	1,132,387
CHS	472	35,917	1,487,112	1,061,114
FHS	2493	37,910	1,661,479	1,130,951
LBC1921	469	36,370	1,629,809	1,139,838
LBC1936	953	36,370	1,629,809	1,137,922
MAP	751	36,342	1,621,490	1,117,217
ORCADES	419	36,346	1,626,425	1,133,261
ROS	687	36,342	1,621,471	1,131,184
**Replication Study**	**Size**	**Mapped Genes**	**SNPs Mapped to Genes**	**Independent SNPs Mapped to Genes**
Sydney MAS	887	36,370	2,402,745	244,827
WGHS	3542	37,978	2,446,788	274,512
NHS-CGEM	2066	36,370	2,401,155	242,854
NHS-GLAU	179	36,370	2,399,007	243,580
NHS-KS	80	36,370	2,390,963	242,325
NHS-T2D	756	36,370	2,402,404	243,998
NHS-ENDO	141	36,370	2,395,954	242,932
NHS-CHD	358	36,370	2,400,863	244,042

Table S2: Sample size and the number of SNPs in the word list delayed recall GWAS from each discovery and replication cohort

Discovery Study	Size	Mapped Genes	SNPs Mapped to Genes	Independent SNPs Mapped to Genes
AGES	2616	35,489	1,543,364	1,096,628
ARIC	9188	36,370	1,629,725	989,308
CHS	334	35,917	1,497,718	976,227
ERF	1267	36,370	1,629,276	1,138,098
GENOA	758	36,270	1,566,203	1,102,863
HBCS	888	36,056	1,557,853	1,100,825
KORCULA	472	36,347	1,621,910	1,133,344
MAP	751	36,342	1,621,490	1,131,751
ROS	687	36,342	1,621,471	1,129,369
RS1	2067	36,352	1,629,532	1,137,296
RS2	1533	36,353	1,628,710	1,131,348
RS3	1935	36,366	1,628,825	1,136,731
SHIP	1474	36,475	1,742,022	1,129,014
SPLIT	303	36,370	1,628,392	1,141,061
TASCOG	331	36,358	1,604,242	1,126,345
**Replication Study**	**Size**	**Mapped Genes**	**SNPs Mapped to Genes**	**Independent SNPs Mapped to Genes**
Sydney MAS	880	36,370	2,402,745	244,262
WGHS	3542	37,978	2,446,788	274,347
NHS-CGEM	1773	36,370	2,400,711	244,310
NHS-GLAU	166	36,370	2,398,566	243,137
NHS-KS	76	36,370	2,390,073	242,090
NHS-T2D	655	36,370	2,402,114	244,131
NHS-ENDO	119	36,369	2,393,809	242,911
NHS-CHD	307	36,370	2,400,354	243,518

Table S3: Tissue-specific relationships between delayed recall test (PAR-dr and WL-dr) summary SNP associations and eQTLs and meQTLs

Tissue	Trait	Summary GWAS	Beta (LogOR)	S.E.	Z	raw_pVal	perm_pVal
All tissues combined	PAR-dr	Discovery	0.52	0.01	38.02	0	<0.001
		Joint Discovery and Replication	0.41	0.01	42.52	0	<0.001
	WL-dr	Discovery	0.37	0.02	23.62	2.50E-123	<0.001
		Joint Discovery and Replication	0.36	0.01	38.70	0	<0.001
adiposeSubcut	PAR-dr	Discovery	0.55	0.02	27.30	4.74E-164	<0.001
		Joint Discovery and Replication	0.45	0.01	31.30	5.06E-215	<0.001
	WL-dr	Discovery	0.42	0.02	18.60	3.45E-77	<0.001
		Joint Discovery and Replication	0.40	0.01	28.28	6.99E-176	<0.001
adiposeVisceral	PAR-dr	Discovery	0.56	0.02	25.22	2.52E-140	<0.001
		Joint Discovery and Replication	0.45	0.02	28.57	1.51E-179	<0.001
	WL-dr	Discovery	0.44	0.02	17.88	1.80E-71	<0.001
		Joint Discovery and Replication	0.41	0.02	26.45	3.53E-154	<0.001
adrenalGland	PAR-dr	Discovery	0.58	0.02	24.96	1.59E-137	<0.001
		Joint Discovery and Replication	0.48	0.02	28.71	3.07E-181	<0.001
	WL-dr	Discovery	0.46	0.03	17.87	1.92E-71	<0.001
		Joint Discovery and Replication	0.45	0.02	27.85	1.08E-170	<0.001
arteryAorta	PAR-dr	Discovery	0.57	0.02	27.10	9.06E-162	<0.001
		Joint Discovery and Replication	0.46	0.02	30.60	1.39E-205	<0.001
	WL-dr	Discovery	0.43	0.02	18.18	6.82E-74	<0.001
		Joint Discovery and Replication	0.43	0.01	29.08	6.19E-186	<0.001
arteryCoronary	PAR-dr	Discovery	0.59	0.02	25.71	9.63E-146	<0.001
		Joint Discovery and Replication	0.49	0.02	29.87	4.32E-196	<0.001
	WL-dr	Discovery	0.45	0.03	17.66	9.21E-70	<0.001
		Joint Discovery and Replication	0.42	0.02	25.98	9.11E-149	<0.001
arteryTibial		Discovery	0.58	0.02	28.20	6.26E-175	<0.001

	PAR-dr	Joint Discovery and Replication	0.46	0.01	31.33	1.70E-215	<0.001
	WL-dr	Discovery	0.40	0.02	17.32	3.34E-67	<0.001
		Joint Discovery and Replication	0.40	0.01	28.14	2.90E-174	<0.001
brainAnCinCortex	PAR-dr	Discovery	0.61	0.03	23.02	3.25E-117	<0.001
		Joint Discovery and Replication	0.51	0.02	26.93	1.07E-159	<0.001
	WL-dr	Discovery	0.48	0.03	16.10	2.61E-58	<0.001
		Joint Discovery and Replication	0.44	0.02	23.48	7.19E-122	<0.001
brainCaudate	PAR-dr	Discovery	0.60	0.02	24.72	6.33E-135	<0.001
		Joint Discovery and Replication	0.50	0.02	28.78	3.90E-182	<0.001
	WL-dr	Discovery	0.43	0.03	15.60	7.87E-55	<0.001
		Joint Discovery and Replication	0.43	0.02	25.33	1.41E-141	<0.001
brainCerebelHemi	PAR-dr	Discovery	0.62	0.02	25.18	6.48E-140	<0.001
		Joint Discovery and Replication	0.51	0.02	28.61	5.29E-180	<0.001
	WL-dr	Discovery	0.46	0.03	16.54	2.05E-61	<0.001
		Joint Discovery and Replication	0.44	0.02	25.47	4.27E-143	<0.001
brainCerebellum	PAR-dr	Discovery	0.60	0.02	25.36	6.54E-142	<0.001
		Joint Discovery and Replication	0.51	0.02	30.10	5.16E-199	<0.001
	WL-dr	Discovery	0.44	0.03	16.50	3.55E-61	<0.001
		Joint Discovery and Replication	0.43	0.02	25.56	4.61E-144	<0.001
brainCortex	PAR-dr	Discovery	0.59	0.02	24.47	3.03E-132	<0.001
		Joint Discovery and Replication	0.49	0.02	28.12	5.84E-174	<0.001
	WL-dr	Discovery	0.44	0.03	15.72	1.08E-55	<0.001
		Joint Discovery and Replication	0.42	0.02	24.68	1.64E-134	<0.001
brainFrontCortex	PAR-dr	Discovery	0.60	0.03	23.91	2.28E-126	<0.001
		Joint Discovery and Replication	0.50	0.02	27.17	1.39E-162	<0.001
	WL-dr	Discovery	0.47	0.03	16.52	2.69E-61	<0.001
		Joint Discovery and Replication	0.43	0.02	23.93	1.50E-126	<0.001
brainHippocampus	PAR-dr	Discovery	0.61	0.03	23.27	9.42E-120	<0.001
		Joint Discovery and Replication	0.52	0.02	27.89	3.83E-171	<0.001
		Discovery	0.45	0.03	15.04	4.31E-51	<0.001

	WL-dr	Joint Discovery and Replication	0.44	0.02	23.88	4.85E-126	<0.001
brainHypothalamus	PAR-dr	Discovery	0.61	0.03	23.59	4.40E-123	<0.001
		Joint Discovery and Replication	0.52	0.02	27.47	4.59E-166	<0.001
	WL-dr	Discovery	0.47	0.03	15.93	3.94E-57	<0.001
		Joint Discovery and Replication	0.43	0.02	23.27	8.94E-120	<0.001
brainNucAccumbens	PAR-dr	Discovery	0.59	0.03	23.40	4.33E-121	<0.001
		Joint Discovery and Replication	0.50	0.02	27.96	5.02E-172	<0.001
	WL-dr	Discovery	0.44	0.03	15.37	2.43E-53	<0.001
		Joint Discovery and Replication	0.43	0.02	24.46	3.63E-132	<0.001
brainPutamen	PAR-dr	Discovery	0.61	0.03	23.71	3.13E-124	<0.001
		Joint Discovery and Replication	0.50	0.02	27.28	6.65E-164	<0.001
	WL-dr	Discovery	0.45	0.03	15.65	3.34E-55	<0.001
		Joint Discovery and Replication	0.44	0.02	24.49	2.13E-132	<0.001
breastMamTissue	PAR-dr	Discovery	0.56	0.02	25.58	2.38E-144	<0.001
		Joint Discovery and Replication	0.46	0.02	29.19	2.73E-187	<0.001
	WL-dr	Discovery	0.43	0.02	17.52	9.65E-69	<0.001
		Joint Discovery and Replication	0.40	0.02	26.00	4.91E-149	<0.001
xformedlymphocytes	PAR-dr	Discovery	0.58	0.03	22.21	2.58E-109	<0.001
		Joint Discovery and Replication	0.50	0.02	26.76	9.33E-158	<0.001
	WL-dr	Discovery	0.47	0.03	16.38	2.45E-60	<0.001
		Joint Discovery and Replication	0.42	0.02	23.37	8.00E-121	<0.001
xformedfibroblasts	PAR-dr	Discovery	0.55	0.02	25.17	8.00E-140	<0.001
		Joint Discovery and Replication	0.45	0.02	28.66	1.33E-180	<0.001
	WL-dr	Discovery	0.45	0.02	18.38	1.82E-75	<0.001
		Joint Discovery and Replication	0.40	0.02	26.34	6.40E-153	<0.001
colonSigmoid	PAR-dr	Discovery	0.58	0.02	25.05	1.56E-138	<0.001
		Joint Discovery and Replication	0.50	0.02	30.38	8.85E-203	<0.001
	WL-dr	Discovery	0.46	0.03	18.06	6.57E-73	<0.001
		Joint Discovery and Replication	0.44	0.02	27.58	2.09E-167	<0.001
colonTransverse		Discovery	0.58	0.02	25.70	1.24E-145	<0.001

	PAR-dr	Joint Discovery and Replication	0.46	0.02	28.22	3.47E-175	<0.001
	WL-dr	Discovery	0.42	0.03	16.72	9.24E-63	<0.001
		Joint Discovery and Replication	0.42	0.02	26.75	1.13E-157	<0.001
esophagusJunction	PAR-dr	Discovery	0.58	0.02	25.52	1.10E-143	<0.001
		Joint Discovery and Replication	0.49	0.02	30.05	2.30E-198	<0.001
	WL-dr	Discovery	0.45	0.03	17.49	1.63E-68	<0.001
		Joint Discovery and Replication	0.41	0.02	25.72	6.21E-146	<0.001
esophagusMucosa	PAR-dr	Discovery	0.58	0.02	27.44	9.54E-166	<0.001
		Joint Discovery and Replication	0.46	0.02	30.42	3.38E-203	<0.001
	WL-dr	Discovery	0.44	0.02	18.69	5.63E-78	<0.001
		Joint Discovery and Replication	0.41	0.01	27.86	8.07E-171	<0.001
esophagusMuscular	PAR-dr	Discovery	0.56	0.02	27.09	1.16E-161	<0.001
		Joint Discovery and Replication	0.47	0.01	31.73	6.35E-221	<0.001
	WL-dr	Discovery	0.44	0.02	19.37	1.26E-83	<0.001
		Joint Discovery and Replication	0.41	0.01	28.70	4.32E-181	<0.001
heartAtrialAppend	PAR-dr	Discovery	0.57	0.02	25.51	1.62E-143	<0.001
		Joint Discovery and Replication	0.47	0.02	28.88	1.88E-183	<0.001
	WL-dr	Discovery	0.45	0.02	18.29	9.52E-75	<0.001
		Joint Discovery and Replication	0.42	0.02	27.01	1.01E-160	<0.001
heartLeftVentricl	PAR-dr	Discovery	0.57	0.02	24.89	9.56E-137	<0.001
		Joint Discovery and Replication	0.47	0.02	28.40	2.15E-177	<0.001
	WL-dr	Discovery	0.44	0.03	17.51	1.13E-68	<0.001
		Joint Discovery and Replication	0.41	0.02	26.03	2.35E-149	<0.001
Liver	PAR-dr	Discovery	0.59	0.03	21.82	1.35E-105	<0.001
		Joint Discovery and Replication	0.50	0.02	25.93	2.74E-148	<0.001
	WL-dr	Discovery	0.47	0.03	15.93	3.78E-57	<0.001
		Joint Discovery and Replication	0.45	0.02	24.42	1.14E-131	<0.001
Lung	PAR-dr	Discovery	0.55	0.02	25.95	1.60E-148	<0.001
		Joint Discovery and Replication	0.45	0.02	29.93	9.08E-197	<0.001
		Discovery	0.43	0.02	18.49	2.68E-76	<0.001

	WL-dr	Joint Discovery and Replication	0.40	0.01	27.87	5.37E-171	<0.001
muscleSkeletal	PAR-dr	Discovery	0.56	0.02	24.98	9.93E-138	<0.001
		Joint Discovery and Replication	0.46	0.02	28.50	1.13E-178	<0.001
	WL-dr	Discovery	0.42	0.03	16.72	9.58E-63	<0.001
		Joint Discovery and Replication	0.40	0.02	25.46	5.27E-143	<0.001
nerveTibial	PAR-dr	Discovery	0.58	0.02	29.71	6.23E-194	<0.001
		Joint Discovery and Replication	0.47	0.01	33.20	1.25E-241	<0.001
	WL-dr	Discovery	0.42	0.02	19.05	7.16E-81	<0.001
		Joint Discovery and Replication	0.41	0.01	29.49	4.11E-191	<0.001
Ovary	PAR-dr	Discovery	0.59	0.03	23.50	3.82E-122	<0.001
		Joint Discovery and Replication	0.51	0.02	28.31	2.73E-176	<0.001
	WL-dr	Discovery	0.47	0.03	17.08	1.93E-65	<0.001
		Joint Discovery and Replication	0.44	0.02	25.54	6.48E-144	<0.001
Pancreas	PAR-dr	Discovery	0.56	0.02	24.72	7.33E-135	<0.001
		Joint Discovery and Replication	0.47	0.02	28.55	2.97E-179	<0.001
	WL-dr	Discovery	0.44	0.03	17.40	8.80E-68	<0.001
		Joint Discovery and Replication	0.41	0.02	25.90	5.90E-148	<0.001
Pituitary	PAR-dr	Discovery	0.62	0.02	25.53	1.02E-143	<0.001
		Joint Discovery and Replication	0.52	0.02	29.49	3.80E-191	<0.001
	WL-dr	Discovery	0.45	0.03	16.25	2.24E-59	<0.001
		Joint Discovery and Replication	0.44	0.02	25.61	1.31E-144	<0.001
Prostate	PAR-dr	Discovery	0.61	0.02	25.17	7.54E-140	<0.001
		Joint Discovery and Replication	0.50	0.02	28.73	1.82E-181	<0.001
	WL-dr	Discovery	0.48	0.03	17.66	8.37E-70	<0.001
		Joint Discovery and Replication	0.44	0.02	25.52	1.16E-143	<0.001
skinNotExposed	PAR-dr	Discovery	0.57	0.02	26.32	1.12E-152	<0.001
		Joint Discovery and Replication	0.46	0.02	29.63	6.50E-193	<0.001
	WL-dr	Discovery	0.45	0.02	18.62	2.33E-77	<0.001
		Joint Discovery and Replication	0.42	0.01	27.92	1.65E-171	<0.001
skinExposed		Discovery	0.57	0.02	27.47	3.59E-166	<0.001

	PAR-dr	Joint Discovery and Replication	0.45	0.01	30.76	9.18E-208	<0.001
	WL-dr	Discovery	0.44	0.02	19.05	6.29E-81	<0.001
		Joint Discovery and Replication	0.41	0.01	28.26	9.56E-176	<0.001
smallIntestine	PAR-dr	Discovery	0.60	0.03	23.89	4.37E-126	<0.001
		Joint Discovery and Replication	0.51	0.02	28.32	2.04E-176	<0.001
	WL-dr	Discovery	0.48	0.03	17.25	1.13E-66	<0.001
		Joint Discovery and Replication	0.44	0.02	25.30	2.82E-141	<0.001
Spleen	PAR-dr	Discovery	0.58	0.02	24.16	5.77E-129	<0.001
		Joint Discovery and Replication	0.48	0.02	28.33	1.48E-176	<0.001
	WL-dr	Discovery	0.45	0.03	17.06	2.83E-65	<0.001
		Joint Discovery and Replication	0.45	0.02	27.25	1.90E-163	<0.001
Stomach	PAR-dr	Discovery	0.58	0.02	25.54	6.91E-144	<0.001
		Joint Discovery and Replication	0.47	0.02	28.90	1.28E-183	<0.001
	WL-dr	Discovery	0.44	0.03	17.15	6.23E-66	<0.001
		Joint Discovery and Replication	0.41	0.02	25.51	1.71E-143	<0.001
Testis	PAR-dr	Discovery	0.57	0.02	25.18	7.08E-140	<0.001
		Joint Discovery and Replication	0.47	0.02	28.94	3.84E-184	<0.001
	WL-dr	Discovery	0.42	0.03	16.45	7.74E-61	<0.001
		Joint Discovery and Replication	0.40	0.02	25.70	1.25E-145	<0.001
Thyroid	PAR-dr	Discovery	0.55	0.02	27.49	2.57E-166	<0.001
		Joint Discovery and Replication	0.45	0.01	31.29	6.03E-215	<0.001
	WL-dr	Discovery	0.42	0.02	18.91	9.05E-80	<0.001
		Joint Discovery and Replication	0.40	0.01	28.89	1.40E-183	<0.001
Uterus	PAR-dr	Discovery	0.62	0.03	23.90	3.00E-126	<0.001
		Joint Discovery and Replication	0.51	0.02	27.36	9.16E-165	<0.001
	WL-dr	Discovery	0.49	0.03	17.00	7.64E-65	<0.001
		Joint Discovery and Replication	0.45	0.02	24.58	1.89E-133	<0.001
Vagina	PAR-dr	Discovery	0.61	0.03	23.42	2.74E-121	<0.001
		Joint Discovery and Replication	0.51	0.02	27.58	2.20E-167	<0.001

	WL-dr	Discovery	0.48	0.03	16.62	5.17E-62	<0.001
		Joint Discovery and Replication	0.44	0.02	24.15	7.87E-129	<0.001
wholeBlood	PAR-dr	Discovery	0.55	0.02	22.24	1.42E-109	<0.001
		Joint Discovery and Replication	0.44	0.02	24.44	6.80E-132	<0.001
	WL-dr	Discovery	0.43	0.03	15.47	5.89E-54	<0.001
		Joint Discovery and Replication	0.39	0.02	22.72	2.81E-114	<0.001
brainHippocampus2017	PAR-dr	Discovery	0.76	0.10	7.66	1.91E-14	<0.001
		Joint Discovery and Replication	0.60	0.07	8.59	8.42E-18	<0.001
	WL-dr	Discovery	0.41	0.12	3.25	0.00114	0.034
		Joint Discovery and Replication	0.41	0.07	5.47	4.42E-08	<0.001
brainHippocampus2017 methyQTL	PAR-dr	Discovery	0.57	0.02	28.60	6.96E-180	<0.001
		Joint Discovery and Replication	0.47	0.01	33.94	1.50E-252	<0.001
	WL-dr	Discovery	0.45	0.02	21.02	4.32E-98	<0.001
		Joint Discovery and Replication	0.42	0.01	31.68	2.67E-220	<0.001

Table S4: Relationship Between Delayed Recall Summary Gene Associations and Transcription Factor Genes

Transcription Factor Gene	Trait	Summary GWAS	Beta (LogOR)	S.E.	Z	raw_pVal	perm_pVal
AR	PAR-dr	Discovery	0.06	0.2	0.32	0.749	1
		Joint	0.14	0.2	0.73	0.466	1
	WL-dr	Discovery	-0.04	0.21	-0.19	0.846	1
		Joint	0.47	0.17	2.8	0.00513	0.384
BACH1	PAR-dr	Discovery	0.45	0.24	1.83	0.067	0.994
		Joint	0.25	0.27	0.93	0.354	1
	WL-dr	Discovery	-0.69	0.39	-1.76	0.0782	0.997
		Joint	0.41	0.25	1.64	0.101	1
CEBPA	PAR-dr	Discovery	0.22	0.03	8.23	1.93e-16	<0.001
		Joint	0.53	0.03	20.02	3.57e-89	<0.001
	WL-dr	Discovery	0.09	0.03	3.21	0.00133	0.18
		Joint	0.57	0.03	21.77	4.3e-105	<0.001
CTCF	PAR-dr	Discovery	0.17	0.03	6.91	4.79e-12	<0.001
		Joint	0.5	0.03	19.62	1.02e-85	<0.001
	WL-dr	Discovery	0.16	0.03	6.26	3.86e-10	<0.001
		Joint	0.6	0.03	23.05	1.6e-117	<0.001
E2F1	PAR-dr	Discovery	0.2	0.03	6.08	1.18e-09	<0.001
		Joint	0.47	0.03	15.15	7.7e-52	<0.001
	WL-dr	Discovery	0.18	0.03	5.3	1.14e-07	0.001
		Joint	0.54	0.03	17.47	2.56e-68	<0.001
E2F4	PAR-dr	Discovery	0.12	0.03	3.59	0.00033	0.076
		Joint	0.32	0.03	10.39	2.73e-25	<0.001
	WL-dr	Discovery	0.12	0.03	3.82	0.000134	0.052
		Joint	0.38	0.03	12.6	2.03e-3	<0.001
EGR1	PAR-dr	Discovery	0.12	0.03	4.49	7.17e-06	0.007
		Joint	0.39	0.03	15.04	3.72e-51	<0.001
	WL-dr	Discovery	0.16	0.03	6	1.93e-09	<0.001
		Joint	0.54	0.03	20.52	1.49e-93	<0.001
EPAS1	PAR-dr	Discovery	0.24	0.13	1.78	0.0756	0.997
		Joint	0.37	0.13	2.93	0.00341	0.364
	WL-dr	Discovery	0.4	0.13	3.21	0.00134	0.18
		Joint	0.63	0.11	5.68	1.31e-08	0.001
ESR1	PAR-dr	Discovery	0.21	0.03	6.81	9.56e-12	<0.001
		Joint	0.46	0.03	15.66	2.88e-55	<0.001
	WL-dr	Discovery	0.13	0.03	4.24	2.25e-05	0.02
		Joint	0.56	0.03	19.31	4.79e-83	<0.001
ETS1	PAR-dr	Discovery	0.12	0.03	4.19	2.82e-05	0.016
		Joint	0.36	0.03	13.39	7.35e-41	<0.001
		Discovery	0.09	0.03	3.33	0.000881	0.143

	WL-dr	Joint	0.41	0.03	15.22	2.45e-52	<0.001
FOS	PAR-dr	Discovery	0.21	0.03	7.62	2.56e-14	<0.001
		Joint	0.46	0.03	16.89	5.5e-64	<0.001
	WL-dr	Discovery	0.13	0.03	4.58	4.73e-06	0.01
		Joint	0.52	0.03	18.98	2.54e-80	<0.001
FOXA1	PAR-dr	Discovery	0.23	0.03	8.88	6.73e-19	<0.001
		Joint	0.56	0.03	21.27	2.15e-100	<0.001
	WL-dr	Discovery	0.15	0.03	5.51	3.49e-08	0.001
		Joint	0.65	0.03	24.54	5.77e-133	<0.001
FOXP1	PAR-dr	Discovery	0.17	0.04	4.09	4.4e-05	0.023
		Joint	0.38	0.04	9.52	1.73e-21	<0.001
	WL-dr	Discovery	0.07	0.04	1.57	0.117	1
		Joint	0.33	0.04	8.29	1.16e-16	<0.001
GATA2	PAR-dr	Discovery	0.27	0.03	8.31	9.63e-17	<0.001
		Joint	0.56	0.03	18.63	1.84e-77	<0.001
	WL-dr	Discovery	0.13	0.03	4.05	5.21e-05	0.036
		Joint	0.6	0.03	19.94	1.73e-88	<0.001
GATA3	PAR-dr	Discovery	0.2	0.03	6.95	3.65e-12	<0.001
		Joint	0.53	0.03	18.76	1.59e-78	<0.001
	WL-dr	Discovery	0.1	0.03	3.51	0.00045	0.101
		Joint	0.6	0.03	21.38	2.03e-101	<0.001
HIF1A	PAR-dr	Discovery	0.27	0.08	3.31	0.000921	0.135
		Joint	0.44	0.08	5.7	1.17e-08	<0.001
	WL-dr	Discovery	0.13	0.09	1.46	0.144	1
		Joint	0.36	0.08	4.59	4.38e-06	0.005
HNF1A	PAR-dr	Discovery	0.07	0.5	0.14	0.89	1
		Joint	0.53	0.4	1.33	0.184	1
	WL-dr	Discovery	0.76	0.35	2.15	0.0316	0.921
		Joint	0.43	0.42	1.02	0.306	1
HNF4A	PAR-dr	Discovery	0.19	0.03	7.03	2.03e-12	<0.001
		Joint	0.46	0.03	17.18	3.99e-66	<0.001
	WL-dr	Discovery	0.12	0.03	4.31	1.64e-05	0.017
		Joint	0.52	0.03	19.58	2.33e-85	<0.001
IGF1R	PAR-dr	Discovery	0.2	0.22	0.91	0.364	1
		Joint	0.31	0.21	1.48	0.139	1
	WL-dr	Discovery	0.07	0.23	0.32	0.749	1
		Joint	0.23	0.22	1.07	0.286	1
JUN	PAR-dr	Discovery	-0.87	0.82	-1.06	0.289	1
		Joint	0.03	0.59	0.06	0.955	1
	WL-dr	Discovery	0.23	0.54	0.44	0.661	1
		Joint	0.39	0.5	0.79	0.432	1
MITF		Discovery	0.19	0.03	5.85	4.91e-09	<0.001

	PAR-dr	Joint	0.46	0.03	15.32	6.07e-53	<0.001
	WL-dr	Discovery	0.11	0.03	3.31	0.000931	0.146
		Joint	0.52	0.03	17.21	2.4e-66	<0.001
NFE2L2	PAR-dr	Discovery	0.49	0.53	0.92	0.357	1
		Joint	-0.36	0.77	-0.46	0.642	1
	WL-dr	Discovery	0.15	0.62	0.24	0.809	1
		Joint	-0.44	0.79	-0.55	0.58	1
PRDM14	PAR-dr	Discovery	0.23	0.04	6.04	1.52e-09	<0.001
		Joint	0.46	0.04	12.98	1.68e-38	<0.001
	WL-dr	Discovery	0.17	0.04	4.42	1.01e-05	0.012
		Joint	0.6	0.03	17.26	1.02e-66	<0.001
RARA	PAR-dr	Discovery	-0.68	0.47	-1.44	0.151	1
		Joint	-1.01	0.52	-1.93	0.0531	0.983
	WL-dr	Discovery	0.11	0.36	0.3	0.762	1
		Joint	-0.39	0.44	-0.88	0.377	1
RB1	PAR-dr	Discovery	0.1	0.03	3.37	0.000741	0.115
		Joint	0.38	0.03	13.54	9.29e-42	<0.001
	WL-dr	Discovery	0.13	0.03	4.52	6.24e-06	0.011
		Joint	0.47	0.03	16.74	6.33e-63	<0.001
RBL2	PAR-dr	Discovery	0.18	0.03	6.92	4.38e-12	<0.001
		Joint	0.48	0.03	18.4	1.42e-75	<0.001
	WL-dr	Discovery	0.11	0.03	4.13	3.62e-05	0.03
		Joint	0.49	0.03	18.74	2.56e-78	<0.001
REST	PAR-dr	Discovery	0.12	0.08	1.57	0.115	1
		Joint	0.42	0.07	6.02	1.72e-09	<0.001
	WL-dr	Discovery	0.24	0.07	3.22	0.00128	0.172
		Joint	0.53	0.07	8.05	8.5e-16	<0.001
RUNX1	PAR-dr	Discovery	0.29	0.04	6.51	7.69e-11	<0.001
		Joint	0.57	0.04	14.06	6.79e-45	<0.001
	WL-dr	Discovery	0.2	0.05	4.45	8.77e-06	0.012
		Joint	0.57	0.04	14.11	3.22e-45	<0.001
SMARCA4	PAR-dr	Discovery	0.19	0.03	7.29	3.16e-13	<0.001
		Joint	0.51	0.03	19.94	1.99e-88	<0.001
	WL-dr	Discovery	0.09	0.03	3.64	0.000272	0.071
		Joint	0.58	0.03	22.51	3.17e-112	<0.001
SOX2	PAR-dr	Discovery	0.32	0.04	8.28	1.25e-16	<0.001
		Joint	0.59	0.04	16.32	7.21e-60	<0.001
	WL-dr	Discovery	0.18	0.04	4.33	1.48e-05	0.016
		Joint	0.61	0.04	16.92	3.22e-64	<0.001
SP1	PAR-dr	Discovery	-0.13	0.35	-0.38	0.705	1
		Joint	0.11	0.31	0.34	0.738	1
		Discovery	-0.32	0.38	-0.86	0.389	1

	WL-dr	Joint	0.24	0.3	0.83	0.408	1
SPI1	PAR-dr	Discovery	0.23	0.04	5.46	4.89e-08	<0.001
		Joint	0.53	0.04	13.91	5.24e-44	<0.001
	WL-dr	Discovery	0.15	0.04	3.45	0.000558	0.114
		Joint	0.58	0.04	15.23	2.14e-52	<0.001
STAT1	PAR-dr	Discovery	0.22	0.03	8.14	3.84e-16	<0.001
		Joint	0.53	0.03	20.4	1.7e-92	<0.001
	WL-dr	Discovery	0.14	0.03	5.21	1.91e-07	0.002
		Joint	0.61	0.03	23.15	1.31e-118	<0.001
TAL1	PAR-dr	Discovery	0.3	0.04	7.61	2.77e-14	<0.001
		Joint	0.58	0.04	15.66	2.63e-55	<0.001
	WL-dr	Discovery	0.21	0.04	5.2	2.02e-07	0.002
		Joint	0.61	0.04	16.46	6.67e-61	<0.001
TBP	PAR-dr	Discovery	-0.61	0.46	-1.34	0.18	1
		Joint	0.15	0.34	0.43	0.666	1
	WL-dr	Discovery	-0.01	0.36	-0.01	0.989	1
		Joint	0.24	0.32	0.74	0.457	1
TFAP2C	PAR-dr	Discovery	0.2	0.03	7.83	4.83e-15	<0.001
		Joint	0.51	0.03	19.59	1.95e-85	<0.001
	WL-dr	Discovery	0.16	0.03	6.26	3.81e-10	<0.001
		Joint	0.58	0.03	22.32	2.53e-110	<0.001
TP53	PAR-dr	Discovery	0.27	0.04	6.11	1e-09	<0.001
		Joint	0.49	0.04	11.93	8.09e-33	<0.001
	WL-dr	Discovery	0.19	0.05	4.08	4.43e-05	0.031
		Joint	0.55	0.04	13.51	1.44e-41	<0.001
TRIM28	PAR-dr	Discovery	0.2	0.04	4.84	1.3e-06	<0.001
		Joint	0.49	0.04	13.04	7.31e-39	<0.001
	WL-dr	Discovery	0.19	0.04	4.59	4.37e-06	0.01
		Joint	0.53	0.04	14.15	1.87e-45	<0.001
VDR	PAR-dr	Discovery	0.18	0.07	2.75	0.00597	0.448
		Joint	0.37	0.06	6.08	1.22e-09	<0.001
	WL-dr	Discovery	0.08	0.07	1.2	0.231	1
		Joint	0.35	0.06	5.71	1.11e-08	0.001
YY1	PAR-dr	Discovery	-0.23	0.3	-0.78	0.432	1
		Joint	0.07	0.26	0.28	0.778	1
	WL-dr	Discovery	0.27	0.24	1.15	0.25	1
		Joint	0.54	0.21	2.57	0.0101	0.577
ZNF263	PAR-dr	Discovery	0.05	0.04	1.22	0.222	1
		Joint	0.24	0.04	5.71	1.15e-08	<0.001
	WL-dr	Discovery	0.14	0.04	3.28	0.00105	0.155
		Joint D	0.4	0.04	10.16	2.9e-24	<0.001
hsa-let-7a-5p		Discovery	-0.39	0.75	-0.52	0.603	1

	PAR-dr	Joint	0.49	0.51	0.96	0.338	1
	WL-dr	Discovery	-1.24	0.98	-1.26	0.208	1
		Joint	0.26	0.57	0.46	0.642	1
hsa-miR-1	PAR-dr	Discovery	-0.09	0.45	-0.2	0.839	1
		Joint	-0.13	0.46	-0.27	0.784	1
	WL-dr	Discovery	0.28	0.38	0.74	0.46	1
		Joint	0.64	0.32	2	0.046	0.97
hsa-miR-101-3p	PAR-dr	Discovery	-0.14	0.61	-0.23	0.822	1
		Joint	0.45	0.47	0.96	0.336	1
	WL-dr	Discovery	0.46	0.46	1	0.319	1
		Joint	0.43	0.47	0.91	0.363	1
hsa-miR-106a-5p	PAR-dr	Discovery	-0.45	0.83	- 0.55	0.586	1
		Joint	0.15	0.65	0.22	0.823	1
	WL-dr	Discovery	-1.45	1.13	-1.28	0.201	1
		Joint	-0.64	0.89	-0.72	0.472	1
hsa-miR-106b-5p	PAR-dr	Discovery	-0.79	0.73	-1.08	0.279	1
		Joint	-0.24	0.6	-0.4	0.692	1
	WL-dr	Discovery	-0.27	0.61	-0.44	0.657	1
		Joint	-0.22	0.59	-0.37	0.713	1
hsa-miR-107	PAR-dr	Discovery	0.58	0.5	1.15	0.251	1
		Joint	0.72	0.47	1.54	0.124	1
	WL-dr	Discovery	0.76	0.45	1.68	0.0928	1
		Joint	0.69	0.47	1.46	0.144	1
hsa-miR-122-5p	PAR-dr	Discovery	-0.02	0.5	-0.04	0.965	1
		Joint	0	0.49	0	0.998	1
	WL-dr	Discovery	-0.04	0.5	-0.07	0.94	1
		Joint	0.24	0.44	0.56	0.579	1
hsa-miR-124-3p	PAR-dr	Discovery	-0.2	0.53	-0.37	0.713	1
		Joint	0.01	0.49	0.03	0.977	1
	WL-dr	Discovery	-0.59	0.62	-0.96	0.336	1
		Joint	0.26	0.44	0.6	0.548	1
hsa-miR-125b-5p	PAR-dr	Discovery	0.08	0.44	0.19	0.853	1
		Joint	0.09	0.44	0.19	0.846	1
	WL-dr	Discovery	0.54	0.36	1.52	0.13	1
		Joint	0.66	0.33	1.97	0.0485	0.976
hsa-miR-130a-3p	PAR-dr	Discovery	0.83	0.44	1.89	0.0584	0.99
		Joint	0.89	0.42	2.12	0.034	0.922
	WL-dr	Discovery	-0.6	0.84	-0.72	0.471	1
		Joint	0.22	0.6	0.36	0.718	1
hsa-miR-133b	PAR-dr	Discovery	0.87	0.43	2.04	0.0414	0.959
		Joint	0.77	0.45	1.7	0.0887	1
		Discovery	-0.11	0.7	-0.16	0.875	1

	WL-dr	Joint	0.57	0.5	1.13	0.257	1
hsa-miR-137	PAR-dr	Discovery	-0.34	0.76	-0.45	0.653	1
		Joint	-0.29	0.75	-0.39	0.7	1
	WL-dr	Discovery	-0.44	0.79	-0.56	0.576	1
		Joint	-0.19	0.72	-0.27	0.787	1
hsa-miR-138-5p	PAR-dr	Discovery	0.45	0.45	0.98	0.326	1
		Joint	0.73	0.39	1.86	0.0623	0.99
	WL-dr	Discovery	-0.44	0.67	-0.67	0.505	1
		Joint	0.06	0.55	0.11	0.915	1
hsa-miR-141-3p	PAR-dr	Discovery	0.07	0.47	0.15	0.884	1
		Joint	0.6	0.36	1.68	0.0923	1
	WL-dr	Discovery	-0.6	0.6	-0.99	0.323	1
		Joint	0.02	0.48	0.04	0.967	1
hsa-miR-145-5p	PAR-dr	Discovery	-0.15	0.34	-0.43	0.668	1
		Joint	0.42	0.26	1.6	0.11	1
	WL-dr	Discovery	0.16	0.3	0.54	0.588	1
		Joint	0.42	0.27	1.59	0.113	1
hsa-miR-155-5p	PAR-dr	Discovery	0.11	0.19	0.61	0.543	1
		Joint	0.66	0.14	4.66	3.22e-06	0.004
	WL-dr	Discovery	0.16	0.18	0.87	0.386	1
		Joint	0.49	0.16	3.17	0.00155	0.185
hsa-miR-15a-5p	PAR-dr	Discovery	-0.54	0.69	-0.78	0.437	1
		Joint	0.47	0.45	1.06	0.288	1
	WL-dr	Discovery	-0.49	0.68	-0.73	0.467	1
		Joint	-0.22	0.61	-0.36	0.719	1
hsa-miR-16-5p	PAR-dr	Discovery	-1.29	0.77	-1.69	0.0914	1
		Joint	0.14	0.46	0.31	0.757	1
	WL-dr	Discovery	0.16	0.47	0.34	0.736	1
		Joint	-0.45	0.6	-0.75	0.451	1
hsa-miR-17-5p	PAR-dr	Discovery	-0.36	0.44	-0.8	0.421	1
		Joint	-0.02	0.39	-0.04	0.967	1
	WL-dr	Discovery	-0.36	0.45	-0.8	0.422	1
		Joint	0.12	0.37	0.33	0.739	1
hsa-miR-181a-5p	PAR-dr	Discovery	-0.04	0.55	-0.07	0.944	1
		Joint	0.47	0.43	1.08	0.28	1
	WL-dr	Discovery	0.09	0.52	0.17	0.867	1
		Joint	0.13	0.51	0.25	0.799	1
hsa-miR-181b-5p	PAR-dr	Discovery	-1.32	1.01	-1.32	0.188	1
		Joint	-0.7	0.83	-0.84	0.399	1
	WL-dr	Discovery	-0.64	0.82	-0.78	0.433	1
		Joint	0.36	0.54	0.66	0.507	1

hsa-miR-182-5p	PAR-dr	Discovery	-0.3	0.78	-0.38	0.704	1
		Joint	0.32	0.6	0.53	0.594	1
	WL-dr	Discovery	-0.79	0.93	-0.85	0.397	1
		Joint	-0.64	0.89	-0.72	0.469	1
hsa-miR-192-5p	PAR-dr	Discovery	-0.48	0.8	- 0.59	0.553	1
		Joint	-0.25	0.74	-0.34	0.734	1
	WL-dr	Discovery	-0.46	0.8	-0.58	0.563	1
		Joint	-0.41	0.78	-0.52	0.6	1
hsa-miR-195-5p	PAR-dr	Discovery	-3.29	1.55	-2.12	0.0339	0.932
		Joint	-1.56	1.07	-1.46	0.145	1
	WL-dr	Discovery	-0.25	0.71	-0.36	0.719	1
		Joint	-0.21	0.7	-0.3	0.765	1
hsa-miR-19a-3p	PAR-dr	Discovery	-0.12	0.56	-0.21	0.836	1
		Joint	0	0.53	-0.01	0.994	1
	WL-dr	Discovery	0.17	0.49	0.36	0.722	1
		Joint	0.5	0.42	1.2	0.23	1
hsa-miR-19b-3p	PAR-dr	Discovery	-0.06	0.71	-0.08	0.935	1
		Joint	0.23	0.63	0.37	0.713	1
	WL-dr	Discovery	0.22	0.63	0.35	0.723	1
		Joint	0.84	0.45	1.86	0.0632	0.997
hsa-miR-200a-3p	PAR-dr	Discovery	0.34	0.48	0.72	0.472	1
		Joint	0.28	0.49	0.58	0.563	1
	WL-dr	Discovery	0.1	0.54	0.18	0.857	1
		Joint	0.35	0.48	0.73	0.466	1
hsa-miR-200b-3p	PAR-dr	Discovery	0.59	0.41	1.45	0.147	1
		Joint	0.51	0.43	1.2	0.231	1
	WL-dr	Discovery	0.25	0.48	0.52	0.601	1
		Joint	0.35	0.46	0.75	0.453	1
hsa-miR-200c-3p	PAR-dr	Discovery	0.49	0.36	1.36	0.174	1
		Joint	0.59	0.35	1.71	0.0874	1
	WL-dr	Discovery	0.08	0.45	0.18	0.859	1
		Joint	0.32	0.4	0.8	0.425	1
hsa-miR-203a	PAR-dr	Discovery	-0.6	0.7	-0.85	0.396	1
		Joint	-1.42	0.9	-1.57	0.115	1
	WL-dr	Discovery	-0.56	0.72	-0.78	0.435	1
		Joint	-0.68	0.75	-0.91	0.361	1
hsa-miR-204-5p	PAR-dr	Discovery	0.08	0.43	0.18	0.859	1
		Joint	0.47	0.35	1.34	0.182	1
	WL-dr	Discovery	-0.07	0.46	-0.14	0.886	1
		Joint	0.42	0.36	1.16	0.247	1
hsa-miR-205-5p	PAR-dr	Discovery	-2.14	1.19	-1.8	0.0716	0.995
		Joint	-0.7	0.8	-0.87	0.382	1
		Discovery	0.45	0.5	0.9	0.37	1

	WL-dr	Joint	0.85	0.4	2.15	0.0319	0.91
hsa-miR-20a-5p	PAR-dr	Discovery	-0.38	0.55	-0.7	0.484	1
		Joint	0.14	0.44	0.31	0.754	1
	WL-dr	Discovery	-0.24	0.52	-0.46	0.646	1
		Joint	-0.07	0.49	-0.15	0.88	1
hsa-miR-20b-5p	PAR-dr	Discovery	0.13	0.66	0.2	0.845	1
		Joint	0.72	0.49	1.48	0.14	1
	WL-dr	Discovery	-2.09	1.33	-1.58	0.114	1
		Joint	0.2	0.64	0.31	0.759	1
hsa-miR-21-5p	PAR-dr	Discovery	-0.51	0.45	-1.13	0.26	1
		Joint	0.23	0.33	0.69	0.491	1
	WL-dr	Discovery	-0.22	0.4	-0.55	0.583	1
		Joint	-0.12	0.39	-0.3	0.761	1
hsa-miR-218-5p	PAR-dr	Discovery	0.27	0.55	0.49	0.624	1
		Joint	0.62	0.45	1.36	0.173	1
	WL-dr	Discovery	1.33	0.3	4.48	7.63e-06	0.011
		Joint	0.73	0.43	1.69	0.0902	1
hsa-miR-221-3p	PAR-dr	Discovery	0.79	0.38	2.09	0.0362	0.942
		Joint	0.86	0.36	2.39	0.0167	0.738
	WL-dr	Discovery	-0.36	0.64	-0.55	0.581	1
		Joint	-0.13	0.59	-0.22	0.827	1
hsa-miR-222-3p	PAR-dr	Discovery	-0.03	0.62	-0.04	0.965	1
		Joint	0.1	0.59	0.18	0.86	1
	WL-dr	Discovery	-0.44	0.73	-0.6	0.552	1
		Joint	-0.26	0.68	-0.37	0.708	1
hsa-miR-223-3p	PAR-dr	Discovery	0.18	0.53	0.33	0.742	1
		Joint	0.7	0.41	1.72	0.0849	1
	WL-dr	Discovery	-0.01	0.58	-0.02	0.986	1
		Joint	-0.57	0.72	-0.79	0.43	1
hsa-miR-23a-3p	PAR-dr	Discovery	0.3	0.54	0.56	0.578	1
		Joint	0.4	0.51	0.78	0.434	1
	WL-dr	Discovery	-0.29	0.69	-0.42	0.674	1
		Joint	0.62	0.45	1.36	0.174	1
hsa-miR-24-3p	PAR-dr	Discovery	-0.06	0.57	-0.1	0.92	1
		Joint	0.31	0.48	0.65	0.518	1
	WL-dr	Discovery	0.5	0.44	1.14	0.253	1
		Joint	0.48	0.45	1.07	0.284	1
hsa-miR-26a-5p	PAR-dr	Discovery	-0.18	0.5	-0.36	0.716	1
		Joint	-0.27	0.51	-0.52	0.604	1
	WL-dr	Discovery	-1.72	0.8	-2.14	0.0328	0.925
		Joint	-0.38	0.54	-0.71	0.477	1
hsa-miR-27a-3p		Discovery	0.01	0.64	0.01	0.988	1

	PAR-dr	Joint	-0.03	0.65	-0.05	0.964	1
	WL-dr	Discovery	-0.43	0.76	-0.57	0.57	1
		Joint	-0.18	0.69	-0.27	0.79	1
hsa-miR-29a-3p	PAR-dr	Discovery	-0.46	0.55	-0.84	0.403	1
		Joint	0.28	0.41	0.7	0.483	1
	WL-dr	Discovery	0.37	0.4	0.95	0.344	1
		Joint	0.92	0.29	3.14	0.0017	0.193
hsa-miR-29b-3p	PAR-dr	Discovery	-0.11	0.43	-0.24	0.808	1
		Joint	0.13	0.39	0.34	0.736	1
	WL-dr	Discovery	0.15	0.39	0.38	0.702	1
		Joint	0.75	0.28	2.63	0.00866	0.528
hsa-miR-29c-3p	PAR-dr	Discovery	-0.31	0.56	-0.56	0.577	1
		Joint	0.28	0.43	0.63	0.526	1
	WL-dr	Discovery	0.19	0.45	0.43	0.67	1
		Joint	0.94	0.3	3.13	0.00172	0.194
hsa-miR-30a-5p	PAR-dr	Discovery	-1.82	1.24	-1.46	0.143	1
		Joint	-1.16	1.04	-1.11	0.266	1
	WL-dr	Discovery	0.34	0.59	0.57	0.569	1
		Joint	0.41	0.57	0.71	0.48	1
hsa-miR-31-5p	PAR-dr	Discovery	0.73	0.33	2.25	0.0246	0.861
		Joint	0.58	0.36	1.63	0.103	1
	WL-dr	Discovery	0.37	0.4	0.95	0.344	1
		Joint	0.53	0.37	1.44	0.149	1
hsa-miR-34a-5p	PAR-dr	Discovery	-0.62	0.51	-1.21	0.225	1
		Joint	-0.34	0.46	-0.73	0.465	1
	WL-dr	Discovery	-0.11	0.42	-0.27	0.789	1
		Joint	-0.39	0.47	-0.84	0.401	1
hsa-miR-424-5p	PAR-dr	Discovery	-0.32	0.62	-0.52	0.603	1
		Joint	-0.24	0.6	-0.4	0.691	1
	WL-dr	Discovery	-1.16	0.82	-1.42	0.155	1
		Joint	-0.28	0.61	-0.45	0.649	1
hsa-miR-7-5p	PAR-dr	Discovery	0.53	0.54	0.98	0.327	1
		Joint	0.99	0.41	2.41	0.016	0.723
	WL-dr	Discovery	0.92	0.43	2.15	0.0316	0.921
		Joint	0.99	0.41	2.4	0.0166	0.741
hsa-miR-9-5p	PAR-dr	Discovery	0.01	0.59	0.01	0.988	1
		Joint	0.36	0.5	0.71	0.48	1
	WL-dr	Discovery	0.49	0.47	1.05	0.296	1
		Joint	0.16	0.55	0.29	0.774	1

NOTE: Joint equals the summary results from meta-analysis of discovery and replication cohorts

Table S5: Significant Genes Associated with Paragraph Delayed Recall (PAR-dr) and Word List Delayed Recall (WL-dr)

Gene Id[1]	Gene Name	chr	start (bp)	end (bp)	strand	Gene_p (PAR-dr)[2]	Gene_p (WL-dr)[3]	Significant[4]
3107	*HLA-C*	6	31236526	31239913	-	3.91E-11	8.73E-18	BOTH
116935	*RPL3P2*	6	31248068	31249348	+	3.91E-11	8.73E-18	BOTH
100287272	*USP8P1*	6	31243351	31246528	+	3.91E-11	8.73E-18	BOTH
352961	*HCG26*	6	31439006	31440185	+	3.91E-11	1.17E-15	BOTH
404026	*MTCO3P1*	6	32673921	32674580	-	3.91E-11	9.64E-14	BOTH
6992	*PPP1R11*	6	30034932	30038110	+	3.91E-11	2.32E-10	BOTH
7726	*TRIM26*	6	30152232	30181271	-	3.91E-11	2.32E-10	BOTH
100421582	*PAIP1P1*	6	30154575	30156383	-	3.91E-11	2.32E-10	BOTH
80352	*RNF39*	6	30038043	30043628	-	3.91E-11	7.42E-09	BOTH
100507679	*MUC22*	6	30973729	31003179	+	5.98E-09	3.05E-20	BOTH
352963	*HLA-P*	6	29767821	29770856	+	5.98E-09	8.73E-18	BOTH
353013	*RPL7AP7*	6	29770910	29771797	-	5.98E-09	8.73E-18	BOTH
353014	*HCG4P9*	6	29766201	29767903	-	5.98E-09	8.73E-18	BOTH
554223	*LOC554223*	6	29759683	29765584	+	5.98E-09	8.73E-18	BOTH
10866	*HCP5*	6	31430957	31433586	+	5.98E-09	1.17E-15	BOTH
11074	*TRIM31*	6	30070674	30080867	-	5.98E-09	1.17E-15	BOTH
267016	*HLA-X*	6	31429623	31430267	-	5.98E-09	1.17E-15	BOTH
352967	*MICG*	6	29780167	29780469	-	5.98E-09	1.17E-15	BOTH
353010	*3.8-1.5*	6	29732893	29734073	-	5.98E-09	1.17E-15	BOTH
3109	*HLA-DMB*	6	32902406	32908847	-	5.98E-09	9.64E-14	BOTH
3116	*HLA-DPB2*	6	33080293	33096890	+	5.98E-09	5.53E-12	BOTH
3120	*HLA-DQB2*	6	32723837	32731330	-	5.98E-09	5.53E-12	BOTH
56244	*BTNL2*	6	32362513	32374900	-	5.98E-09	5.53E-12	BOTH
267013	*HLA-DPA3*	6	33098974	33099120	-	5.98E-09	5.53E-12	BOTH
3118	*HLA-DQA2*	6	32709156	32714664	+	5.98E-09	2.32E-10	BOTH
10107	*TRIM10*	6	30119722	30128711	-	5.98E-09	2.32E-10	BOTH
10255	*HCG9*	6	29942892	29946180	+	5.98E-09	2.32E-10	BOTH
79897	*RPP21*	6	30312897	30314635	+	5.98E-09	2.32E-10	BOTH
89870	*TRIM15*	6	30130968	30140473	+	5.98E-09	2.32E-10	BOTH
135644	*TRIM40*	6	30103885	30116512	+	5.98E-09	2.32E-10	BOTH
285829	*SUMO2P1*	6	29603231	29604281	-	5.98E-09	2.32E-10	BOTH
414778	*HCG17*	6	30201816	30293911	-	5.98E-09	2.32E-10	BOTH
30834	*ZNRD1*	6	30029017	30032686	+	5.98E-09	7.42E-09	BOTH
56658	*TRIM39*	6	30294227	30311506	+	5.98E-09	7.42E-09	BOTH
414777	*HCG18*	6	30255174	30294933	-	5.98E-09	7.42E-09	BOTH
4855	*NOTCH4*	6	32162620	32191844	-	4.01E-07	3.05E-20	BOTH
100507436	*MICA*	6	31367561	31383090	+	4.01E-07	3.05E-20	BOTH

54435	*HCG4*	6	29758808	29760850	-	4.01E-07	8.73E-18	BOTH
352962	*HLA-V*	6	29759530	29760527	+	4.01E-07	8.73E-18	BOTH
3106	*HLA-B*	6	31321649	31324989	-	4.01E-07	1.17E-15	BOTH
3135	*HLA-G*	6	29794756	29798899	+	4.01E-07	1.17E-15	BOTH
9659	*PDE4DIP*	1	14485142 4	145076186	-	4.01E-07	1.17E-15	BOTH
267017	*HLA-Z*	6	32864179	32864266	+	4.01E-07	1.17E-15	BOTH
340198	*IFITM4P*	6	29718584	29718925	-	4.01E-07	1.17E-15	BOTH
353000	*HCGVIII-2*	6	29801426	29802705	-	4.01E-07	1.17E-15	BOTH
353005	*HCG4P8*	6	29793906	29794891	-	4.01E-07	1.17E-15	BOTH
729816	*DHFRP2*	6	31331244	31334742	-	4.01E-07	1.17E-15	BOTH
100129192	*MICC*	6	30382490	30387543	+	4.01E-07	1.17E-15	BOTH
100294145	*LOC100294145*	6	32861953	32871535	+	4.01E-07	1.17E-15	BOTH
353002	*HCG4P4*	6	29922982	29923410	-	4.01E-07	9.64E-14	BOTH
394214	*COL11A2P1*	6	33071470	33074921	-	4.01E-07	9.64E-14	BOTH
414764	*HCG23*	6	32358287	32361468	+	4.01E-07	9.64E-14	BOTH
3108	*HLA-DMA*	6	32916391	32920900	-	4.01E-07	5.53E-12	BOTH
3139	*HLA-L*	6	30227339	30234728	+	4.01E-07	5.53E-12	BOTH
4279	*MICD*	6	29938149	29943522	-	4.01E-07	5.53E-12	BOTH
387117	*UBQLN1P1*	6	30326371	30331888	-	4.01E-07	5.53E-12	BOTH
404024	*TRIM26BP*	6	30206078	30210056	+	4.01E-07	5.53E-12	BOTH
3137	*HLA-J*	6	29973748	29977733	+	4.01E-07	2.32E-10	BOTH
6824	*ETF1P1*	6	29999490	30001654	+	4.01E-07	2.32E-10	BOTH
267014	*HLA-N*	6	30318851	30319815	+	4.01E-07	2.32E-10	BOTH
353001	*HCG4P3*	6	29972622	29973605	-	4.01E-07	2.32E-10	BOTH
100507399	*HCG8*	6	29979878	29981699	-	4.01E-07	2.32E-10	BOTH
135656	*DPCR1*	6	30908777	30921998	+	4.01E-07	7.42E-09	BOTH
80862	*ZNRD1-AS1*	6	29968788	30028961	-	4.01E-07	1.83E-07	BOTH
442184	*OR2B3*	6	29053985	29055090	-	4.01E-07	1.83E-07	BOTH
442185	*OR2J1*	6	29068720	29069655	+	4.01E-07	1.83E-07	BOTH
26717	*OR2G1P*	6	29197007	29197997	-	4.01E-07	3.52E-06	PAR
442189	*OR2H4P*	6	29183013	29183953	+	4.01E-07	3.52E-06	PAR
56940	*DUSP22*	6	292101	351355	+	4.01E-07	5.28E-05	PAR
285834	*HCG22*	6	31021227	31027667	+	1.54E-05	8.73E-18	WL
100131609	*HNRNPA1P2*	6	32293175	32294298	+	1.54E-05	8.73E-18	WL
3122	*HLA-DRA*	6	32407619	32412823	+	1.54E-05	1.17E-15	WL
5460	*POU5F1*	6	31132114	31138451	-	1.54E-05	1.17E-15	WL
267015	*HLA-S*	6	31349346	31350264	-	1.54E-05	1.17E-15	WL
100462812	*FGFR3P1*	6	31345494	31345805	+	1.54E-05	1.17E-15	WL
1460	*CSNK2B*	6	31632995	31637844	+	1.54E-05	9.64E-14	WL
3132	*HLA-DRB9*	6	32427597	32427866	-	1.54E-05	9.64E-14	WL

7917	*BAG6*	6	31606805	31620953	-	1.54E-05	9.64E-14	WL
7918	*GPANK1*	6	31629006	31634060	-	1.54E-05	9.64E-14	WL
55937	*APOM*	6	31620187	31625987	+	1.54E-05	9.64E-14	WL
57827	*C6ORF47*	6	31626075	31628549	-	1.54E-05	9.64E-14	WL
79969	*ATAT1*	6	30594613	30614598	+	1.54E-05	9.64E-14	WL
253018	*HCG27*	6	31165537	31171745	+	1.54E-05	9.64E-14	WL
353019	*HCG9P5*	6	29716031	29716425	+	1.54E-05	9.64E-14	WL
394263	*MUC21*	6	30951485	30957675	+	1.54E-05	9.64E-14	WL
100130889	*PSORS1C3*	6	31141512	31145676	-	1.54E-05	9.64E-14	WL
2550	*GABBR1*	6	29570005	29600962	-	1.54E-05	5.53E-12	WL
3113	*HLA-DPA1*	6	33032346	33048555	-	1.54E-05	5.53E-12	WL
3115	*HLA-DPB1*	6	33043703	33057473	+	1.54E-05	5.53E-12	WL
6163	*RPL32P1*	6	33047076	33047788	+	1.54E-05	5.53E-12	WL
7916	*PRRC2A*	6	31588450	31605554	+	1.54E-05	5.53E-12	WL
7920	*ABHD16A*	6	31654726	31671137	-	1.54E-05	5.53E-12	WL
10537	*UBD*	6	29523389	29527702	-	1.54E-05	5.53E-12	WL
58530	*LY6G6D*	6	31683048	31685698	+	1.54E-05	5.53E-12	WL
79136	*LY6G6E*	6	31679753	31681842	-	1.54E-05	5.53E-12	WL
81696	*OR5V1*	6	29323007	29324054	-	1.54E-05	5.53E-12	WL
259215	*LY6G6F*	6	31674625	31678372	+	1.54E-05	5.53E-12	WL
352964	*HLA-T*	6	29864220	29866724	+	1.54E-05	5.53E-12	WL
352965	*HLA-U*	6	29901541	29902657	+	1.54E-05	5.53E-12	WL
352966	*HLA-W*	6	29923611	29926835	+	1.54E-05	5.53E-12	WL
442191	*OR14J1*	6	29274467	29275432	+	1.54E-05	5.53E-12	WL
442197	*OR2I1P*	6	29520882	29521940	+	1.54E-05	5.53E-12	WL
646702	*HLA-DPA2*	6	33059259	33061091	-	1.54E-05	5.53E-12	WL
3105	*HLA-A*	6	29910247	29913661	+	1.54E-05	2.32E-10	WL
3138	*HLA-K*	6	29894436	29897616	+	1.54E-05	2.32E-10	WL
5758	*PTMAP1*	6	30601227	30603014	+	1.54E-05	2.32E-10	WL
6046	*BRD2*	6	32936437	32949282	+	1.54E-05	2.32E-10	WL
26713	*OR2H5P*	6	29541850	29542428	+	1.54E-05	2.32E-10	WL
58496	*LY6G5B*	6	31638728	31640227	+	1.54E-05	2.32E-10	WL
80741	*LY6G5C*	6	31644461	31648150	-	1.54E-05	2.32E-10	WL
162540	*SPPL2C*	17	43922256	43924438	+	1.54E-05	2.32E-10	WL
285492	*LINC00955*	4	3578596	3592712	+	1.54E-05	2.32E-10	WL
353003	*HCG4P5*	6	29908689	29909578	-	1.54E-05	2.32E-10	WL
80868	*HCG4B*	6	29892369	29894992	-	1.54E-05	7.42E-09	WL
100128977	*MAPT-AS1*	17	43920722	43972879	-	1.54E-05	7.42E-09	WL
26701	*OR2N1P*	6	29105547	29106693	-	1.54E-05	1.83E-07	WL
442181	*RPSAP2*	6	28699716	28700751	+	1.54E-05	1.83E-07	WL
442183	*OR2P1P*	6	29039609	29040343	-	1.54E-05	1.83E-07	WL

646260	*SAR1AP1*	6	29042193	29045018	-	1.54E-05	1.83E-07	WL
10665	*C6ORF10*	6	32260475	32339705	-	0.000371751	8.73E-18	WL
352957	*MICF*	6	29819964	29821829	-	0.000371751	8.73E-18	WL
353009	*3.8-1.4*	6	29833692	29834864	-	0.000371751	8.73E-18	WL
28973	*MRPS18B*	6	30585275	30594174	+	0.000371751	9.64E-14	WL
81797	*OR12D3*	6	29341200	29343068	-	0.000371751	9.64E-14	WL
221545	*C6ORF136*	6	30614816	30620987	+	0.000371751	9.64E-14	WL
353004	*HCG4P7*	6	29853887	29854784	-	0.000371751	9.64E-14	WL
5514	*PPP1R10*	6	30568177	30585084	-	0.000371751	5.53E-12	WL
26529	*OR12D2*	6	29364416	29365448	+	0.000371751	5.53E-12	WL
100507362	*LINC01015*	6	29497183	29501345	+	0.000371751	5.53E-12	WL
5987	*TRIM27*	6	28870779	28891768	-	0.000371751	2.32E-10	WL
8449	*DHX16*	6	30620896	30640830	-	0.000371751	2.32E-10	WL
9292	*GPR53P*	6	29505589	29506428	-	0.000371751	2.32E-10	WL
387043	*RPL13AP*	6	29550285	29550802	+	0.000371751	2.32E-10	WL
414760	*HCG14*	6	28864145	28865099	+	0.000371751	2.32E-10	WL
677820	*SNORA38*	6	31590856	31590987	+	0.000371751	2.32E-10	WL
692092	*SNORD32B*	6	29550029	29550105	+	0.000371751	2.32E-10	WL
353008	*3.8-1.3*	6	29878012	29879177	-	0.000371751	7.42E-09	WL
729583	*C6ORF100*	6	28911561	28912315	+	0.000371751	7.42E-09	WL
9656	*MDC1*	6	30667584	30685458	-	0.000371751	1.83E-07	WL
10211	*FLOT1*	6	30695506	30710687	-	0.000371751	1.83E-07	WL
11270	*NRM*	6	30655824	30659197	-	0.000371751	1.83E-07	WL
26692	*OR2W1*	6	29011990	29012952	-	0.000371751	1.83E-07	WL
26694	*OR2U2P*	6	29236242	29237198	-	0.000371751	1.83E-07	WL
26695	*OR2U1P*	6	29230436	29231856	-	0.000371751	1.83E-07	WL
79313	*OR2AD1P*	6	28994457	28995384	-	0.000371751	1.83E-07	WL
170954	*PPP1R18*	6	30644166	30655672	-	0.000371751	1.83E-07	WL
442190	*OR2B4P*	6	29258373	29259527	+	0.000371751	1.83E-07	WL
646192	*NOP56P1*	6	28751410	28751781	-	0.000371751	1.83E-07	WL
646520	*SUCLA2P1*	6	30436650	30438699	+	0.000371751	1.83E-07	WL
3136	*HLA-H*	6	29855383	29858856	+	0.005788218	9.64E-14	WL
285830	*HLA-F-AS1*	6	29694378	29716826	-	0.005788218	9.64E-14	WL
4280	*MICE*	6	29709234	29716880	-	0.005788218	5.53E-12	WL
7932	*OR2H2*	6	29553818	29556745	+	0.005788218	2.32E-10	WL
80863	*PRRT1*	6	32116140	32119720	-	0.005788218	2.32E-10	WL
152274	*LOC152274*	3	14840838	14852921	-	0.005788218	2.32E-10	WL
353020	*HCG4P11*	6	29688955	29689944	-	0.005788218	2.32E-10	WL
442192	*DDX6P1*	6	29297406	29298838	-	0.005788218	2.32E-10	WL
23	*ABCF1*	6	30539170	30559309	+	0.005788218	7.42E-09	WL
3133	*HLA-E*	6	30457183	30461982	+	0.005788218	7.42E-09	WL

3134	*HLA-F*	6	29691117	29695073	+	0.005788218	7.42E-09	WL
3879	*KRT18P1*	6	28936849	28938244	-	0.005788218	7.42E-09	WL
6148	*RPL23AP1*	6	29694409	29694931	-	0.005788218	7.42E-09	WL
221547	*RANP1*	6	30453662	30454724	+	0.005788218	7.42E-09	WL
414766	*HCG16*	6	28953755	28956399	+	0.005788218	7.42E-09	WL
282890	*ZNF311*	6	28962562	28973037	-	0.005788218	1.83E-07	WL
387044	*RPL13P*	6	28829177	28829793	+	0.005788218	1.83E-07	WL
401242	*LOC401242*	6	28827402	28831454	-	0.005788218	1.83E-07	WL
63940	*GPSM3*	6	32158543	32163300	-	0.05724465	5.53E-12	WL
10554	*AGPAT1*	6	32135983	32145888	-	0.05724465	2.32E-10	WL
177	*AGER*	6	32148745	32152099	-	0.05724465	7.42E-09	WL
5089	*PBX2*	6	32152510	32157963	-	0.05724465	7.42E-09	WL
6048	*RNF5*	6	32146162	32148570	+	0.05724465	7.42E-09	WL
9374	*PPT2*	6	32121229	32131458	+	0.05724465	7.42E-09	WL
80864	*EGFL8*	6	32132382	32136062	+	0.05724465	7.42E-09	WL
4277	*MICB*	6	31462054	31478901	+	0.05724465	1.83E-07	WL
149775	*GNAS-AS1*	20	57393973	57425958	-	0.336579569	1.83E-07	WL

Gene Id[1]: NCBI gene identifier

Gene_p (PAR-dr)[2]: meta-analysis p-value of paragraph delayed recall (PAR-dr)

Gene_p (WL-dr)[3]: meta-analysis p-value of word list delayed recall (WL-dr)

Significant[4] : both PAR-dr and WL-dr are significant after multiple adjustment (BOTH); only PAR-dr is significant (PAR) and only WL-dr is significant (WL)

Table S6. Significant component genes in the six memory-associated pathways

GeneID	Gene	Class	Chr	Start (bp)	End (bp)	Strand	GEO_ID
3105	*HLA-A*	MHC I	6	29910247	29913661	+	GDS4135, GDS4231, GDS4358
3106	*HLA-B*	MHC I	6	31321649	31324989	-	GDS4135, GDS4231, GDS4358
3107	*HLA-C*	MHC I	6	31236526	31239913	-	GDS4135, GDS4231, GDS4358
3133	*HLA-E*	MHC I	6	30457183	30461982	+	GDS4135, GDS4231, GDS4358
3134	*HLA-F*	MHC I	6	29691117	29695073	+	GDS4135, GDS4231, GDS4358
3135	*HLA-G*	MHC I	6	29794756	29798899	+	GDS4135, GDS4231, GDS4358
3136	*HLA-H*	MHC I	6	29855383	29858856	+	
3108	*HLA-DMA*	MHC II	6	32916391	32920900	-	GDS4135, GDS4231, GDS4358
3109	*HLA-DMB*	MHC II	6	32902406	32908847	-	GDS4135, GDS4231, GDS4358
3113	*HLA-DPA1*	MHC II	6	33032346	33048555	-	GDS4135, GDS4231, GDS4358
3115	*HLA-DPB1*	MHC II	6	33043703	33057473	+	GDS4135, GDS4231, GDS4358
3116	*HLA-DPB2*	MHC II	6	33080293	33096890	+	GDS4135, GDS4231, GDS4358
3118	*HLA-DQA2*	MHC II	6	32709156	32714664	+	
3120	*HLA-DQB2*	MHC II	6	32723837	32731330	-	GDS4135, GDS4231, GDS4358
3122	*HLA-DRA*	MHC II	6	32407619	32412823	+	GDS4135, GDS4231, GDS4358

NOTE: This table shows the characteristics of the significant component genes from the six memory-associated pathways. GeneID is the NCBI gene identifier; Gene is the gene name or symbol; Class is the class of the specific MHC gene; chr is the chromosome; start(bp) is the basepair position corresponding to the

beginning of the gene; end(bp) is the basepair position corresponding to the end of the gene; Strand indicates the strand corresponding to the start and end basepair positions; and GEO_ID: the expression data that has the gene measured.

Table S7. Homologous genes in memory-associated pathways for differential expression analysis

Human	Homologous Gene	Gene ID	GEO_ID
**House mouse**			
HLA-A	H2-K1 histocompatibility 2, K1, K region	14972	GDS2082
	H2-D1 histocompatibility 2, D region locus 1	14964	GDS2082
	H2-Bl histocompatibility 2, blastocyst	14963	GDS2082
	H2-Q7 histocompatibility 2, Q region locus 7	15018	GDS2082
	H2-Q10 histocompatibility 2, Q region locus 10	15007	GDS2082
	H2-Q1 histocompatibility 2, Q region locus 1	15006	GDS2082
HLA-G	H2-M3 histocompatibility 2, M region locus 3	14991	GDS2082
HLA-H	Hfe hemochromatosis	15216	GDS2082
HLA-DMA	H2-DMa histocompatibility 2, class II, locus Dma	14998	GDS2082
HLA-DMB	H2-DMb2 histocompatibility 2, class II, locus Mb2	15000	GDS2082
	H2-DMb1 histocompatibility 2, class II, locus Mb1	14999	GDS2082
HLA-DRA	H2-Ea-ps histocompatibility 2, class II antigen E alpha, pseudogene	100504404	GDS2082
**Norway rat**			
HLA-A	RT1-CE5 RT1 class I, locus CE5	309607	GDS2639
	RT1-CE16 RT1 class I, locus CE16	414819	GDS2639, GDS520
HLA-E	RT1-S3 RT1 class Ib, locus S3	294228	GDS2639, GDS520
HLA-G	RT1-M3-1 RT1 class Ib, locus M3, gene 1	24747	GDS2639, GDS520
HLA-H	Hfe hemochromatosis	29199	GDS2639, GDS520
HLA-DRA	RT1-Da RT1 class II, locus Da	294269	GDS2639, GDS520

NOTE: GeneID is the NCBI gene identifier; and GEO_ID: the expression data that has the gene measured.

4. Supplemental Figures

Figure S1: GWAS cohorts and microarray expression datasets

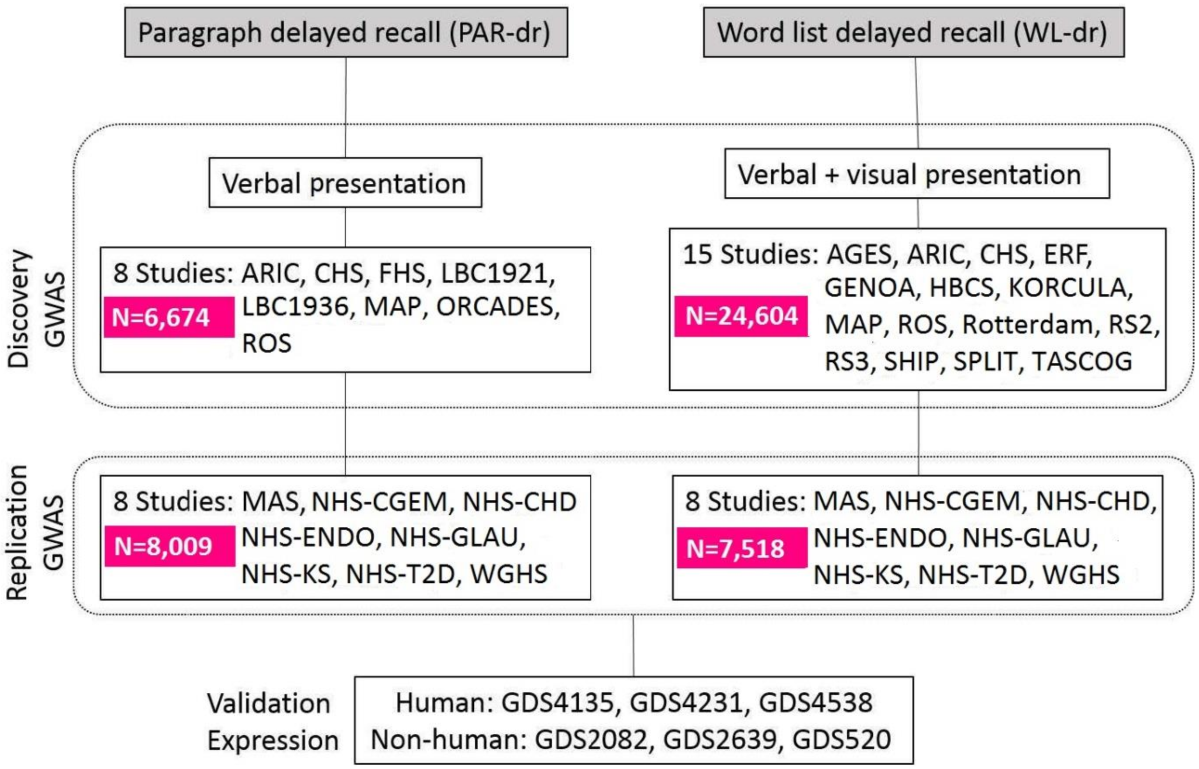

Figure S1. Samples included in the discovery, replication, and differential expression analyses. The discovery pathway analyses included eight cohorts with paragraph delayed recall (PAR-dr) and fifteen cohorts with word list delayed recall (WL-dr). The replication pathway analyses contained eight cohorts for both PAR-dr and WL-dr. The microarray expression studies included three human and three rodent curated data sets from the NCBI Gene Expression Omnibus (GEO). Sample size of each cohort and summaries of the GWAS SNPs are shown in **Tables S1 and S2**.

Figure S2: Design of the pathway analyses

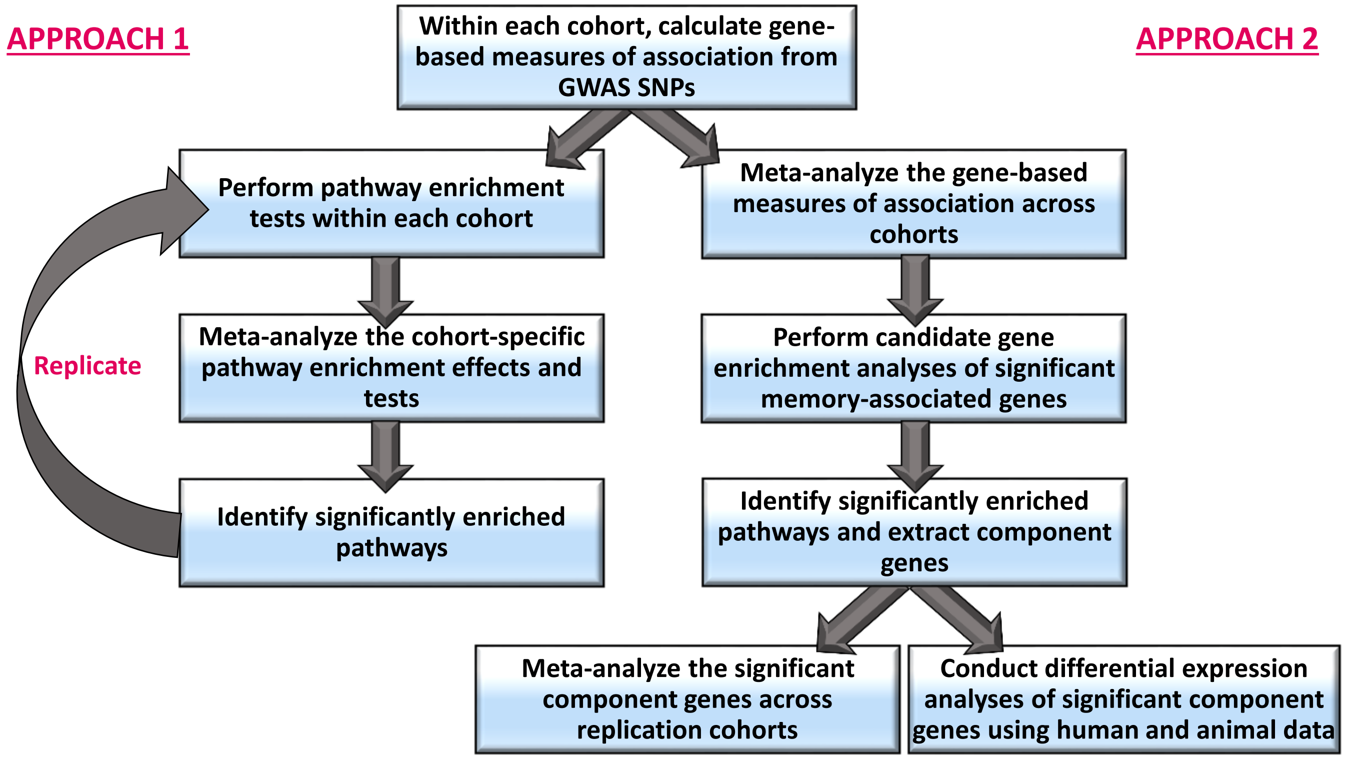

Figure S3. Forest plots of significant pathway enrichment effects and p-values from discovery cohorts (Approach 1)

Pathway: KEGG type 1 diabetes

N: the number of effective genes in the pathway; M: the number of significant genes; and Padj: permutation-adjusted pathway p-value at an individual study.

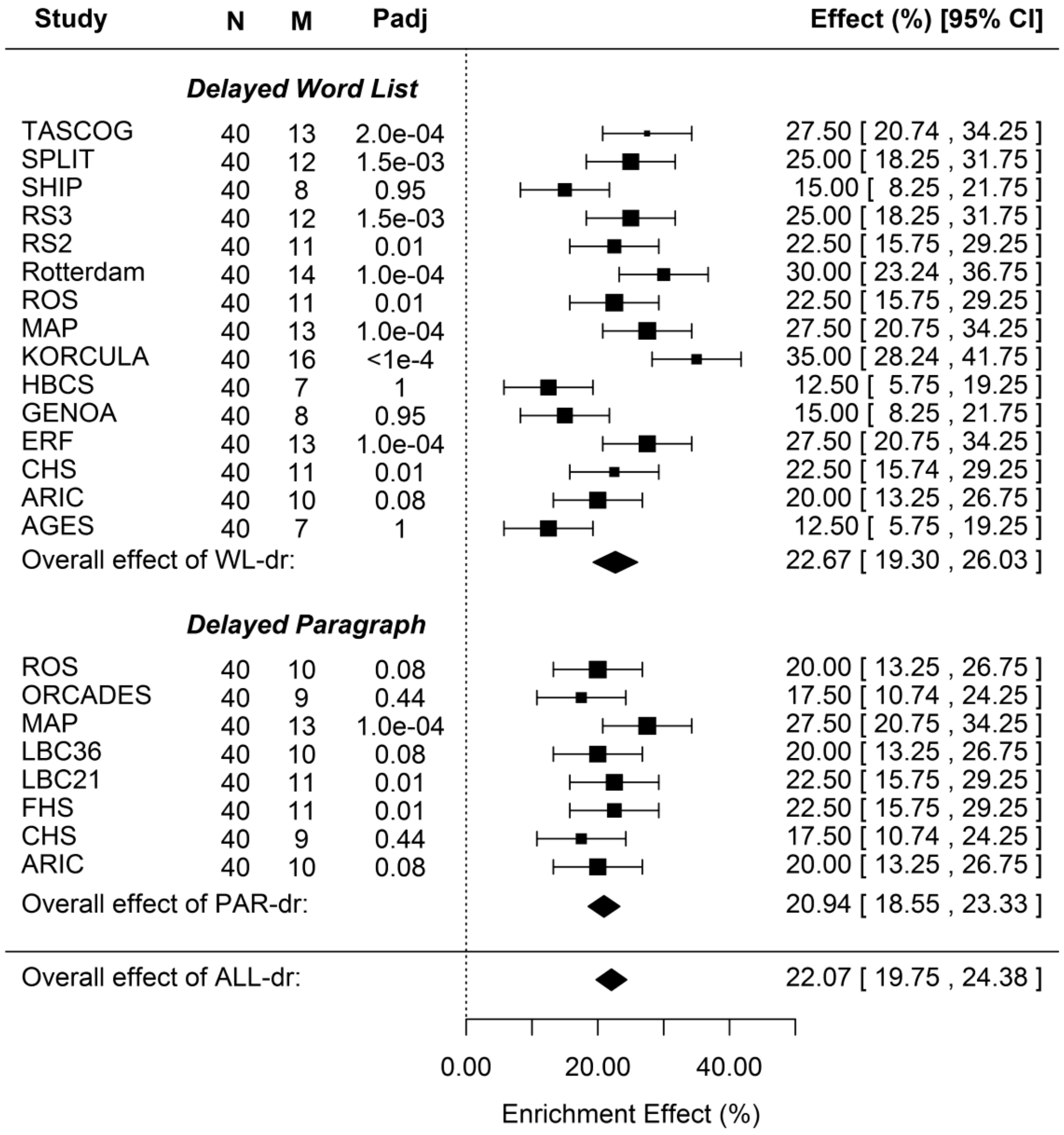

Pathway: PSMD4 targets (snpGeneSets PID 7100)

N: the number of effective genes in the pathway; M: the number of significant genes; and Padj: permutation-adjusted pathway p-value at an individual study.

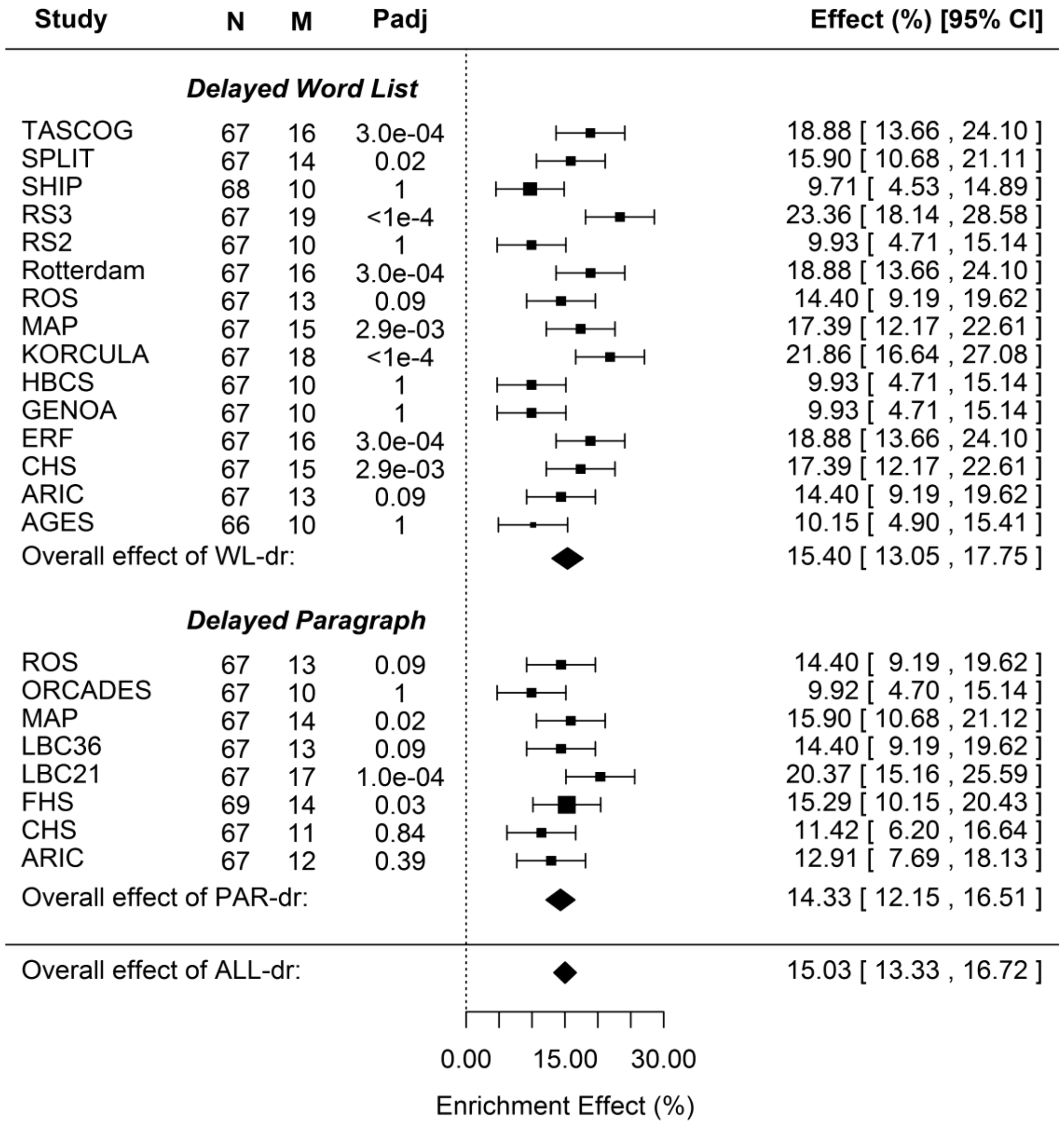

Pathway: KEGG graft-versus-host disease (snpGeneSets PID 2898)

N: the number of effective genes in the pathway; M: the number of significant genes; and Padj: permutation-adjusted pathway p-value at an individual study.

Study	N	M	Padj	Effect (%) [95% CI]
Delayed Word List				
TASCOG	36	14	<1e-4	33.88 [26.76 , 41.01]
SPLIT	36	12	3.0e-04	28.33 [21.22 , 35.45]
SHIP	36	7	1	14.44 [7.32 , 21.56]
RS3	36	12	3.0e-04	28.33 [21.22 , 35.45]
RS2	36	9	0.21	20.00 [12.88 , 27.12]
Rotterdam	36	16	<1e-4	39.44 [32.32 , 46.56]
ROS	36	12	3.0e-04	28.34 [21.22 , 35.45]
MAP	36	13	1.0e-04	31.11 [23.99 , 38.23]
KORCULA	36	14	<1e-4	33.89 [26.77 , 41.01]
HBCS	34	7	1	15.59 [8.26 , 22.91]
GENOA	36	7	1	14.45 [7.33 , 21.56]
ERF	36	13	1.0e-04	31.11 [23.99 , 38.23]
CHS	35	11	2.3e-03	26.43 [19.20 , 33.65]
ARIC	36	10	0.03	22.78 [15.66 , 29.90]
AGES	34	7	1	15.59 [8.26 , 22.91]
Overall effect of WL-dr:				25.60 [21.54 , 29.65]
Delayed Paragraph				
ROS	36	13	1.0e-04	31.11 [23.99 , 38.23]
ORCADES	36	9	0.21	20.00 [12.88 , 27.12]
MAP	36	12	3.0e-04	28.34 [21.22 , 35.45]
LBC36	36	9	0.2	20.00 [12.88 , 27.12]
LBC21	36	12	3.0e-04	28.33 [21.22 , 35.45]
FHS	36	10	0.03	22.78 [15.66 , 29.90]
CHS	35	8	0.71	17.86 [10.63 , 25.08]
ARIC	36	11	4.1e-03	25.56 [18.44 , 32.68]
Overall effect of PAR-dr:				24.26 [20.94 , 27.58]
Overall effect of ALL-dr:				25.13 [22.28 , 27.98]

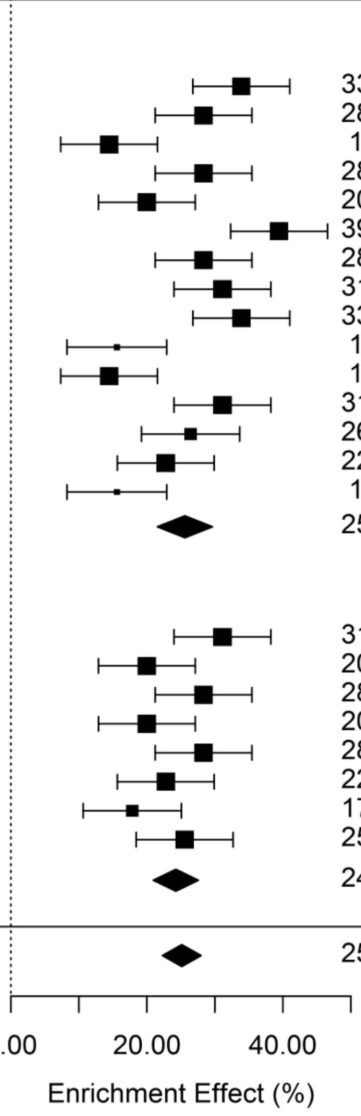

Pathway: KEGG allograft rejection (snpGeneSets PID 2897)

N: the number of effective genes in the pathway; M: the number of significant genes; and Padj: permutation-adjusted pathway p-value at an individual study.

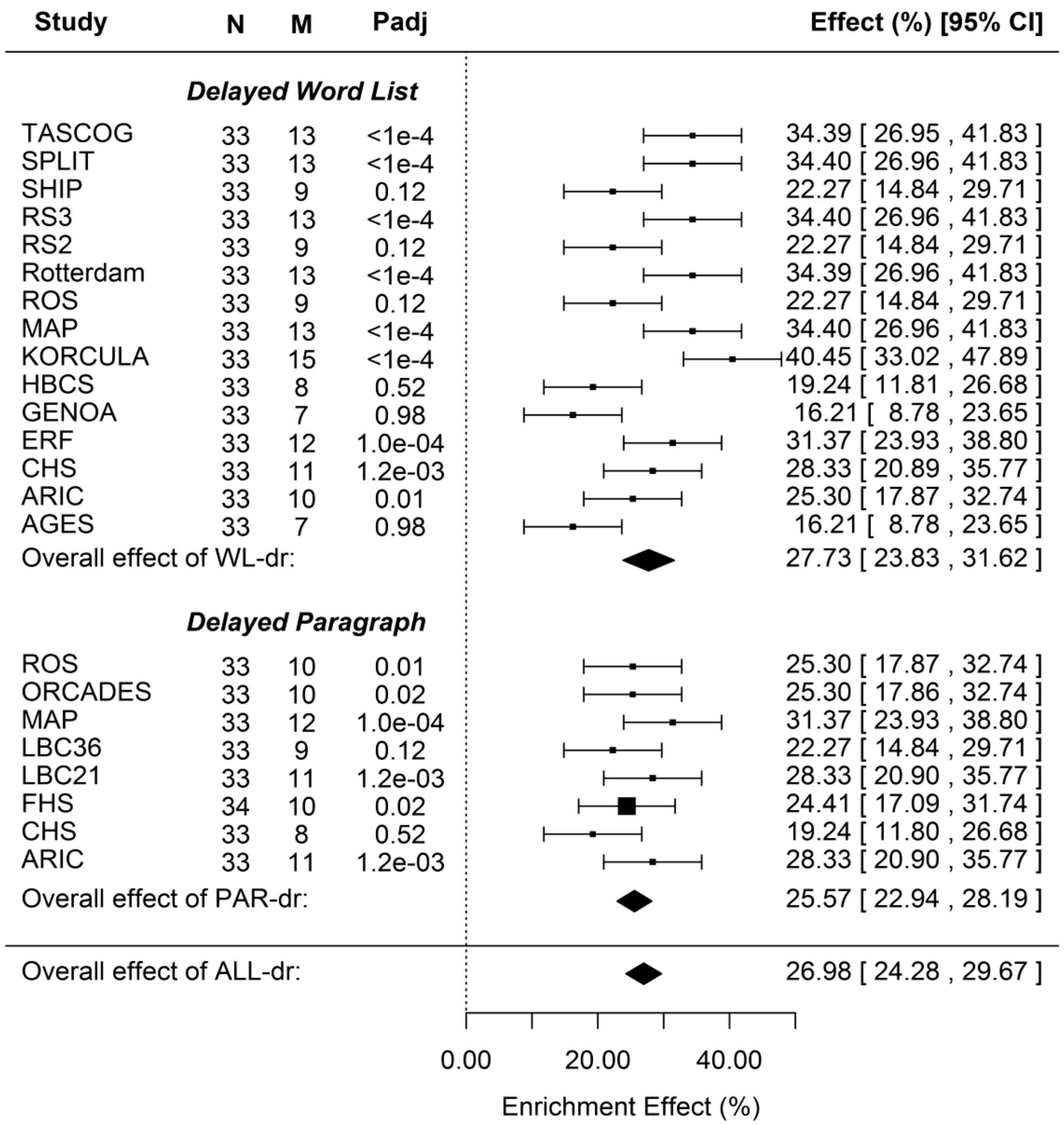

Pathway: KEGG antigen processing and presentation (snpGeneSets PID 2837)

N: the number of effective genes in the pathway; M: the number of significant genes; and Padj: permutation-adjusted pathway p-value at an individual study.

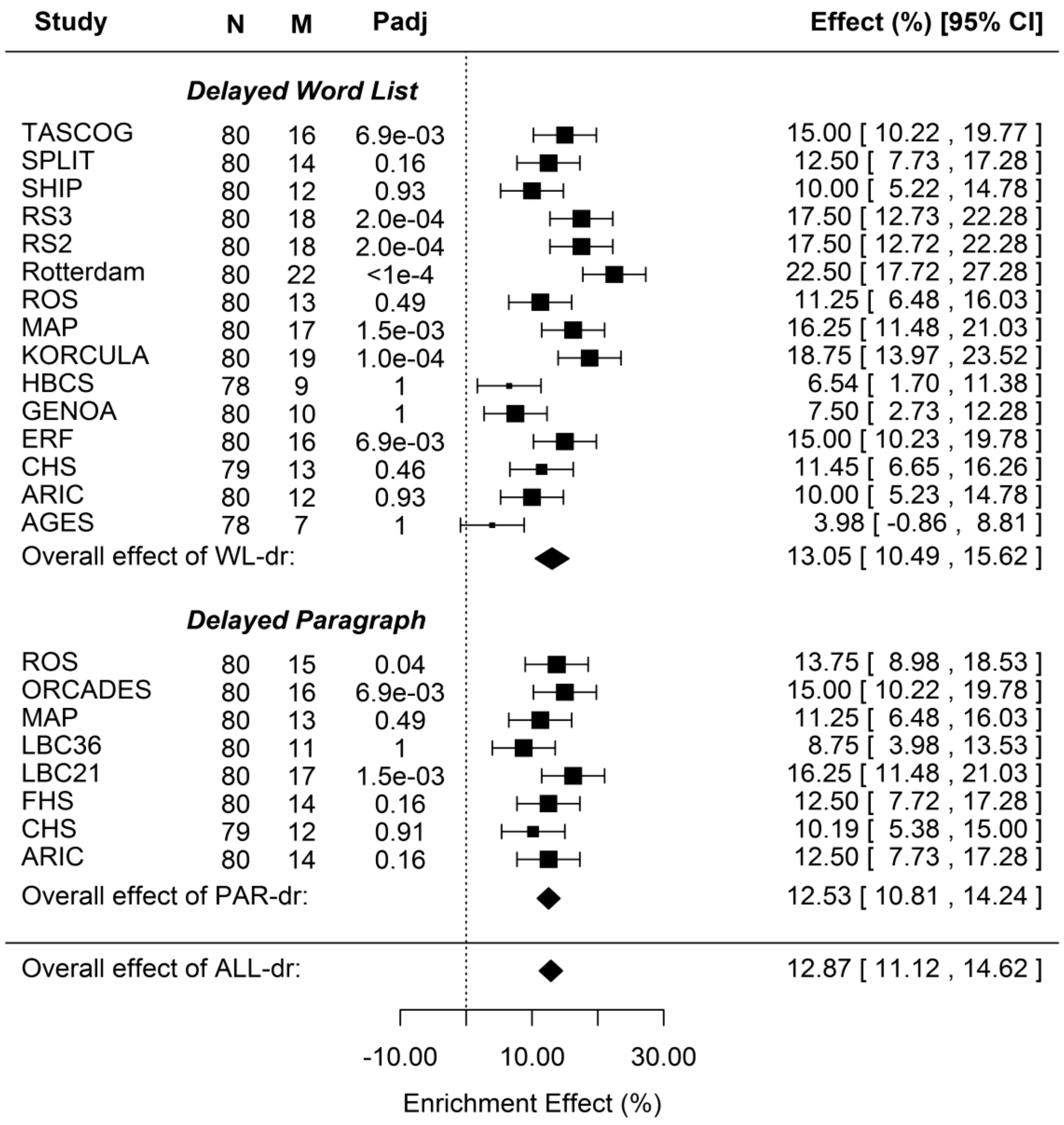

Pathway: KEGG viral myocarditis (snpGeneSets PID 2903)

N: the number of effective genes in the pathway; M: the number of significant genes; and Padj: permutation-adjusted pathway p-value at an individual study.

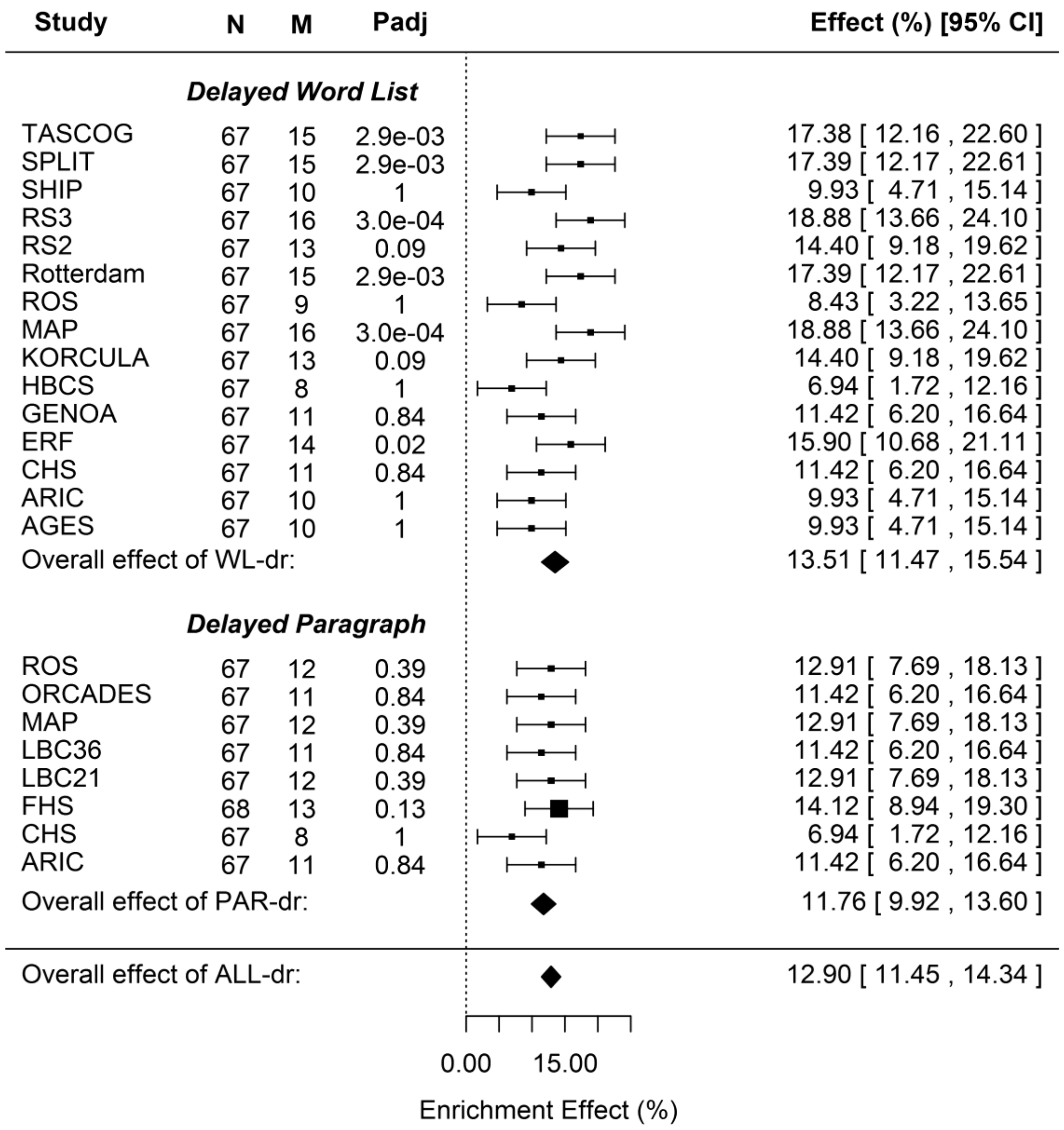

5. References:

1 Psaty, B. M. *et al.* Cohorts for Heart and Aging Research in Genomic Epidemiology (CHARGE) Consortium: Design of prospective meta-analyses of genome-wide association studies from 5 cohorts. *Circulation. Cardiovascular genetics* **2**, 73-80, doi:10.1161/CIRCGENETICS.108.829747 (2009).

2 Debette, S. *et al.* Genome-wide studies of verbal declarative memory in nondemented older people: the Cohorts for Heart and Aging Research in Genomic Epidemiology consortium. *Biological psychiatry* **77**, 749-763, doi:10.1016/j.biopsych.2014.08.027 (2015).

3 The Atherosclerosis Risk in Communities (ARIC) Study: design and objectives. The ARIC investigators. *American journal of epidemiology* **129**, 687-702 (1989).

4 Rosamond, W. D. *et al.* Stroke incidence and survival among middle-aged adults: 9-year follow-up of the Atherosclerosis Risk in Communities (ARIC) cohort. *Stroke* **30**, 736-743 (1999).

5 Mosley, T. H., Jr. *et al.* Cerebral MRI findings and cognitive functioning: the Atherosclerosis Risk in Communities study. *Neurology* **64**, 2056-2062, doi:10.1212/01.WNL.0000165985.97397.88 (2005).

6 Knopman, D. S. & Ryberg, S. A verbal memory test with high predictive accuracy for dementia of the Alzheimer type. *Archives of neurology* **46**, 141-145 (1989).

7 Knopman, D. S., Mosley, T. H., Catellier, D. J., Coker, L. H. & Atherosclerosis Risk in Communities Study Brain, M. R. I. S. Fourteen-year longitudinal study of vascular risk factors, APOE genotype, and cognition: the ARIC MRI Study. *Alzheimer's & dementia : the journal of the Alzheimer's Association* **5**, 207-214, doi:10.1016/j.jalz.2009.01.027 (2009).

8 Lesage, S. R. *et al.* Retinal microvascular abnormalities and cognitive decline: the ARIC 14-year follow-up study. *Neurology* **73**, 862-868, doi:10.1212/WNL.0b013e3181b78436 (2009).

9 Fried, L. P. *et al.* The Cardiovascular Health Study: design and rationale. *Annals of epidemiology* **1**, 263-276 (1991).

10 Longstreth, W. T., Jr. *et al.* Frequency and predictors of stroke death in 5,888 participants in the Cardiovascular Health Study. *Neurology* **56**, 368-375 (2001).

11 Lopez, O. L. *et al.* Evaluation of dementia in the cardiovascular health cognition study. *Neuroepidemiology* **22**, 1-12, doi:67110 (2003).

12 Lopez, O. L. *et al.* Neuropsychological characteristics of mild cognitive impairment subgroups. *J Neurol Neurosurg Psychiatry* **77**, 159-165, doi:10.1136/jnnp.2004.045567 (2006).

13 D, D., J, K. & E, K. *The California Verbal Learning Test-Research Edition*. (New York, NY: Psychological Corporation, 1987).

14 D, W. (New York: Psychological Corporation).

15 Dawber, T. R. & Kannel, W. B. The Framingham study. An epidemiological approach to coronary heart disease. *Circulation* **34**, 553-555 (1966).

16 Feinleib, M., Kannel, W. B., Garrison, R. J., McNamara, P. M. & Castelli, W. P. The Framingham Offspring Study. Design and preliminary data. *Prev Med* **4**, 518-525 (1975).

17 Splansky, G. L. *et al.* The Third Generation Cohort of the National Heart, Lung, and Blood Institute's Framingham Heart Study: design, recruitment, and initial examination. *American journal of epidemiology* **165**, 1328-1335, doi:10.1093/aje/kwm021 (2007).

18 Au, R. *et al.* New norms for a new generation: cognitive performance in the framingham offspring cohort. *Exp Aging Res* **30**, 333-358, doi:10.1080/03610730490484380 (2004).

19 D, W. *Wechsler memory scale*. (Psychological Corporation, 1945).

20 Carandang, R. *et al.* Trends in incidence, lifetime risk, severity, and 30-day mortality of stroke over the past 50 years. *JAMA : the journal of the American Medical Association* **296**, 2939-2946, doi:10.1001/jama.296.24.2939 (2006).

21 Seshadri, S. *et al.* The lifetime risk of stroke: estimates from the Framingham Study. *Stroke* **37**, 345-350, doi:10.1161/01.STR.0000199613.38911.b2 (2006).
22 Deary, I. J. *et al.* The Lothian Birth Cohort 1936: a study to examine influences on cognitive ageing from age 11 to age 70 and beyond. *BMC geriatrics* **7**, 28, doi:10.1186/1471-2318-7-28 (2007).
23 Deary, I. J., Whiteman, M. C., Starr, J. M., Whalley, L. J. & Fox, H. C. The impact of childhood intelligence on later life: following up the Scottish mental surveys of 1932 and 1947. *Journal of personality and social psychology* **86**, 130-147, doi:10.1037/0022-3514.86.1.130 (2004).
24 Deary, I. J., Gow, A. J., Pattie, A. & Starr, J. M. Cohort profile: the Lothian Birth Cohorts of 1921 and 1936. *Int J Epidemiol* **41**, 1576-1584, doi:10.1093/ije/dyr197 (2012).
25 D, W. *WMS-IIIUK administration and scoring manual*. (Psychological Corporation, 1998).
26 Chibnik, L. B. *et al.* CR1 is associated with amyloid plaque burden and age-related cognitive decline. *Annals of neurology* **69**, 560-569, doi:10.1002/ana.22277 (2011).
27 Bennett, D. A. *et al.* Neuropathology of older persons without cognitive impairment from two community-based studies. *Neurology* **66**, 1837-1844, doi:10.1212/01.wnl.0000219668.47116.e6 (2006).
28 Bennett, D. A. *et al.* The Rush Memory and Aging Project: study design and baseline characteristics of the study cohort. *Neuroepidemiology* **25**, 163-175, doi:10.1159/000087446 (2005).
29 Morris, J. C. *et al.* The Consortium to Establish a Registry for Alzheimer's Disease (CERAD). Part I. Clinical and neuropsychological assessment of Alzheimer's disease. *Neurology* **39**, 1159-1165 (1989).
30 McQuillan, R. *et al.* Runs of homozygosity in European populations. *American journal of human genetics* **83**, 359-372, doi:10.1016/j.ajhg.2008.08.007 (2008).
31 Harris, T. B. *et al.* Age, Gene/Environment Susceptibility-Reykjavik Study: multidisciplinary applied phenomics. *American journal of epidemiology* **165**, 1076-1087, doi:10.1093/aje/kwk115 (2007).
32 Palm, W. M. *et al.* Ventricular dilation: association with gait and cognition. *Annals of neurology* **66**, 485-493, doi:10.1002/ana.21739 (2009).
33 Service, S. *et al.* Magnitude and distribution of linkage disequilibrium in population isolates and implications for genome-wide association studies. *Nature genetics* **38**, 556-560, doi:10.1038/ng1770 (2006).
34 Aulchenko, Y. S. *et al.* Linkage disequilibrium in young genetically isolated Dutch population. *European journal of human genetics : EJHG* **12**, 527-534, doi:10.1038/sj.ejhg.5201188 (2004).
35 van Koolwijk, L. M. *et al.* Association of cognitive functioning with retinal nerve fiber layer thickness. *Investigative ophthalmology & visual science* **50**, 4576-4580, doi:10.1167/iovs.08-3181 (2009).
36 A, R. *L'Examen Clinique en Psychologie*. (Presses Universitaires de France, 1964).
37 Lahti, J. *et al.* Glucocorticoid receptor gene haplotype predicts increased risk of hospital admission for depressive disorders in the Helsinki birth cohort study. *J Psychiatr Res* **45**, 1160-1164, doi:10.1016/j.jpsychires.2011.03.008 (2011).
38 Barker, D. J., Osmond, C., Forsen, T. J., Kajantie, E. & Eriksson, J. G. Trajectories of growth among children who have coronary events as adults. *The New England journal of medicine* **353**, 1802-1809, doi:10.1056/NEJMoa044160 (2005).
39 Rudan, I., Campbell, H. & Rudan, P. Genetic epidemiological studies of eastern Adriatic Island isolates, Croatia: objective and strategies. *Coll Antropol* **23**, 531-546 (1999).
40 Rudan, I. *et al.* Effects of inbreeding, endogamy, genetic admixture, and outbreeding on human health: a (1001 Dalmatians) study. *Croatian medical journal* **47**, 601-610 (2006).
41 Campbell, H. *et al.* Effects of genome-wide heterozygosity on a range of biomedically relevant human quantitative traits. *Hum Mol Genet* **16**, 233-241, doi:10.1093/hmg/ddl473 (2007).
42 Polasek, O. *et al.* Genome-wide association study of anthropometric traits in Korcula Island, Croatia. *Croatian medical journal* **50**, 7-16 (2009).

43 Vitart, V. *et al.* Heritabilities of ocular biometrical traits in two croatian isolates with extended pedigrees. *Investigative ophthalmology & visual science* **51**, 737-743, doi:10.1167/iovs.09-3720 (2010).
44 Vitart, V. *et al.* SLC2A9 is a newly identified urate transporter influencing serum urate concentration, urate excretion and gout. *Nature genetics* **40**, 437-442, doi:10.1038/ng.106 (2008).
45 Ikram, M. A. *et al.* Objectives, design and main findings until 2020 from the Rotterdam Study. *Eur J Epidemiol* **35**, 483-517, doi:10.1007/s10654-020-00640-5 (2020).
46 Hofman, A. *et al.* The Rotterdam Study: 2010 objectives and design update. *Eur J Epidemiol* **24**, 553-572, doi:10.1007/s10654-009-9386-z (2009).
47 Hofman, A., Grobbee, D. E., de Jong, P. T. & van den Ouweland, F. A. Determinants of disease and disability in the elderly: the Rotterdam Elderly Study. *Eur J Epidemiol* **7**, 403-422 (1991).
48 Hofman, A. Recent trends in cardiovascular epidemiology. *Eur J Epidemiol* **24**, 721-723, doi:10.1007/s10654-009-9410-3 (2009).
49 Prins, N. D. *et al.* Cerebral small-vessel disease and decline in information processing speed, executive function and memory. *Brain : a journal of neurology* **128**, 2034-2041, doi:10.1093/brain/awh553 (2005).
50 Brand, N. & Jolles, J. Learning and retrieval rate of words presented auditorily and visually. *J Gen Psychol* **112**, 201-210, doi:10.1080/00221309.1985.9711004 (1985).
51 Bots, M. L. *et al.* Prevalence of stroke in the general population. The Rotterdam Study. *Stroke* **27**, 1499-1501 (1996).
52 Hollander, M. *et al.* Incidence, risk, and case fatality of first ever stroke in the elderly population. The Rotterdam Study. *J Neurol Neurosurg Psychiatry* **74**, 317-321 (2003).
53 Volzke, H. *et al.* Cohort profile: the study of health in Pomerania. *Int J Epidemiol* **40**, 294-307, doi:10.1093/ije/dyp394 (2011).
54 Wittchen, H. U., Lachner, G., Wunderlich, U. & Pfister, H. Test-retest reliability of the computerized DSM-IV version of the Munich-Composite International Diagnostic Interview (M-CIDI). *Soc Psychiatry Psychiatr Epidemiol* **33**, 568-578 (1998).
55 Appel, K. *et al.* Moderation of adult depression by a polymorphism in the FKBP5 gene and childhood physical abuse in the general population. *Neuropsychopharmacology* **36**, 1982-1991, doi:10.1038/npp.2011.81 (2011).
56 Callisaya, M. L. *et al.* A population-based study of sensorimotor factors affecting gait in older people. *Age Ageing* **38**, 290-295, doi:10.1093/ageing/afp017 (2009).
57 Martin, K. *et al.* Visuospatial ability and memory are associated with falls risk in older people: a population-based study. *Dement Geriatr Cogn Disord* **27**, 451-457, doi:10.1159/000216840 (2009).
58 M, L. *Neuropsychological testing*. (Oxford University Press, 2004).
59 Sachdev, P. S. *et al.* The Sydney Memory and Ageing Study (MAS): methodology and baseline medical and neuropsychiatric characteristics of an elderly epidemiological non-demented cohort of Australians aged 70-90 years. *Int Psychogeriatr* **22**, 1248-1264, doi:10.1017/S1041610210001067 (2010).
60 D, W. *Wechsler Memory Scale - Third edition*. (The Psychological Corporation, 1997).
61 Ridker, P. M. *et al.* Rationale, design, and methodology of the Women's Genome Health Study: a genome-wide association study of more than 25,000 initially healthy american women. *Clin Chem* **54**, 249-255, doi:10.1373/clinchem.2007.099366 (2008).
62 Kang, J. H. & Grodstein, F. Postmenopausal hormone therapy, timing of initiation, APOE and cognitive decline. *Neurobiology of aging* **33**, 1129-1137, doi:10.1016/j.neurobiolaging.2010.10.007 (2012).
63 Barrett, T. *et al.* NCBI GEO: archive for functional genomics data sets--10 years on. *Nucleic Acids Res* **39**, D1005-1010, doi:10.1093/nar/gkq1184 (2011).

64 Simpson, J. E. *et al.* Microarray analysis of the astrocyte transcriptome in the aging brain: relationship to Alzheimer's pathology and APOE genotype. *Neurobiology of aging* **32**, 1795-1807, doi:10.1016/j.neurobiolaging.2011.04.013 (2011).
65 Borjabad, A. *et al.* Significant effects of antiretroviral therapy on global gene expression in brain tissues of patients with HIV-1-associated neurocognitive disorders. *PLoS pathogens* **7**, e1002213, doi:10.1371/journal.ppat.1002213 (2011).
66 Gelman, B. B. *et al.* The National NeuroAIDS Tissue Consortium brain gene array: two types of HIV-associated neurocognitive impairment. *PLoS One* **7**, e46178, doi:10.1371/journal.pone.0046178 (2012).
67 NCBI HomoloGene. *https://www.ncbi.nlm.nih.gov/homologene*.
68 Verbitsky, M. *et al.* Altered hippocampal transcript profile accompanies an age-related spatial memory deficit in mice. *Learn Mem* **11**, 253-260, doi:10.1101/lm.68204 (2004).
69 Rowe, W. B. *et al.* Hippocampal expression analyses reveal selective association of immediate-early, neuroenergetic, and myelinogenic pathways with cognitive impairment in aged rats. *J Neurosci* **27**, 3098-3110, doi:10.1523/JNEUROSCI.4163-06.2007 (2007).
70 Blalock, E. M. *et al.* Gene microarrays in hippocampal aging: statistical profiling identifies novel processes correlated with cognitive impairment. *J Neurosci* **23**, 3807-3819 (2003).
